# Two Distinct Aerobic Methionine Salvage Pathways Generate Volatile Methanethiol in Rhodopseudomonas palustris

**DOI:** 10.1128/mBio.00407-18

**Published:** 2018-04-10

**Authors:** Anthony R. Miller, Justin A. North, John A. Wildenthal, F. Robert Tabita

**Affiliations:** a Department of Microbiology, The Ohio State University, Columbus, Ohio, USA; Harvard University

**Keywords:** *Rhodopseudomonas palustris*, methanethiol, methionine salvage

## Abstract

5′-Methyl-thioadenosine (MTA) is a dead-end, sulfur-containing metabolite and cellular inhibitor that arises from *S-*adenosyl-l-methionine-dependent reactions. Recent studies have indicated that there are diverse bacterial methionine salvage pathways (MSPs) for MTA detoxification and sulfur salvage. Here, via a combination of gene deletions and directed metabolite detection studies, we report that under aerobic conditions the facultatively anaerobic bacterium Rhodopseudomonas palustris employs both an MTA-isoprenoid shunt identical to that previously described in Rhodospirillum rubrum and a second novel MSP, both of which generate a methanethiol intermediate. The additional R. palustris aerobic MSP, a dihydroxyacetone phosphate (DHAP)-methanethiol shunt, initially converts MTA to 2-(methylthio)ethanol and DHAP. This is identical to the initial steps of the recently reported anaerobic ethylene-forming MSP, the DHAP-ethylene shunt. The aerobic DHAP-methanethiol shunt then further metabolizes 2-(methylthio)ethanol to methanethiol, which can be directly utilized by O-acetyl-l-homoserine sulfhydrylase to regenerate methionine. This is in contrast to the anaerobic DHAP-ethylene shunt, which metabolizes 2-(methylthio)ethanol to ethylene and an unknown organo-sulfur intermediate, revealing functional diversity in MSPs utilizing a 2-(methylthio)ethanol intermediate. When MTA was fed to aerobically growing cells, the rate of volatile methanethiol release was constant irrespective of the presence of sulfate, suggesting a general housekeeping function for these MSPs up through the methanethiol production step. Methanethiol and dimethyl sulfide (DMS), two of the most important compounds of the global sulfur cycle, appear to arise not only from marine ecosystems but from terrestrial ones as well. These results reveal a possible route by which methanethiol might be biologically produced in soil and freshwater environments.

## IMPORTANCE

Biologically available sulfur is often limiting in the environment. Therefore, many organisms have developed methionine salvage pathways (MSPs) to recycle sulfur-containing by-products back into the amino acid methionine. The metabolically versatile bacterium Rhodopseudomonas palustris is unusual in that it possesses two RuBisCOs and two RuBisCO-like proteins. While RuBisCO primarily serves as the carbon fixation enzyme of the Calvin cycle, RuBisCOs and certain RuBisCO-like proteins have also been shown to function in methionine salvage. This work establishes that only one of the R. palustris RuBisCO-like proteins functions as part of an MSP. Moreover, in the presence of oxygen, to salvage sulfur, R. palustris employs two pathways, both of which result in production of volatile methanethiol, a key compound of the global sulfur cycle. When total available sulfur was plentiful, methanethiol was readily released into the environment. However, when sulfur became limiting, methanethiol release decreased, presumably due to methanethiol utilization to regenerate needed methionine.

## INTRODUCTION

**FIG 1:**
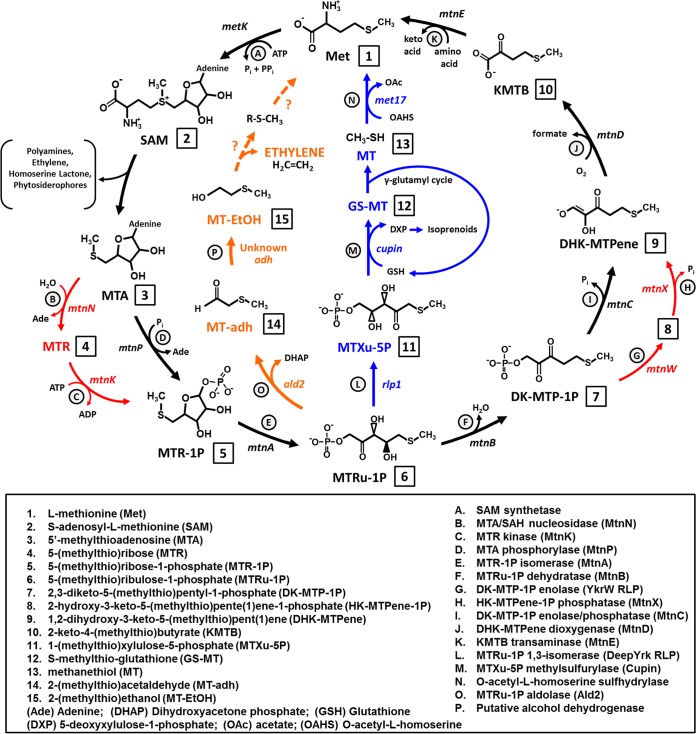
Previously identified bacterial variations on anaerobic and aerobic methionine salvage pathways (see reference 15). (Black) Strictly aerobic canonical (“universal”) MSP from Klebsiella pneumoniae, also found in most eukaryotes (plants, animals, fungi). (Red) *Bacillus* sp. variation in which the MTA phosphorylase (MtnP) is replaced by an MTA nucleosidase (MtnN) and 5-(methylthio)ribose kinase (MtnK) and the bifunctional DK-MTP-1P enolase/phosphatase (MtnC) is replaced by a YkrW clade RLP (MtnW) functioning as a DK-MTP-1P enolase and a separate phosphatase (MtnX). (Blue) Aerobic-anaerobic MTA-isoprenoid shunt MSP in R. rubrum in which a DeepYkr clade RLP (Rlp1) functions as a 5-(methylthio)xylulose-1-P 1,3-isomerase to form 1-(methylthio)xylulose-5-P as a precursor to methanethiol and DXP for isoprenoid biosynthesis. (Orange) Exclusively anaerobic DHAP-ethylene shunt MSP from R. rubrum and R. palustris in which a novel class II aldolase (Ald2) catalyzes cleavage of 5-(methylthio)ribulose-1-P to form DHAP and 2-(methylthio)acetaldehyde as a precursor to ethylene. Compounds are indicated numerically; enzyme names are indicated alphabetically; gene designations are in italics.

In all cells, specific metabolic reactions are essential to maintain intracellular organic sulfur pools. For example, synthesis of sulfur-containing amino acids l-cysteine and l-methionine, coenzymes such as thiamine and coenzyme A, and cosubstrates such as glutathione and *S-*adenosyl-l-methionine (SAM) (Fig. 1; compound 2) requires sulfur in the proper oxidation state. In the environment, the total concentration of sulfur, usually in the form of sulfate, can be quite low, including in such environments as freshwater (~100 μM), anoxic bogs (~20 μM), and flooded soil ecosystems (20 μM to 20 mM) (1–5). Thus, in order to maintain proper intracellular organic sulfur levels, organisms have developed salvage pathways for recycling dead-end sulfur-containing by-products arising from key metabolic reactions. For example, nearly all organisms possess the active methyl cycle (6) to regenerate l-methionine from *S-*adenosyl-l-homocysteine, which is a by-product of SAM-dependent methyltransferase reactions. Similarly, SAM also serves as a cosubstrate for synthesis of polyamines (6), 1-aminocyclopropane-1-carboxylate (the ethylene precursor of plants) (7), acyl- and aryl-homoserine lactone quorum sensing compounds (8), phytosiderophores (9), and certain betaine lipids (10). As a result of these reactions, the inhibitory and dead-end by-product 5′-methyl-thioadenosine (MTA) is formed (Fig. 1; compound 3).

Intracellular buildup of MTA can result in cell cytotoxicity (6). Therefore, nearly all organisms, save some obligate endosymbionts/pathogens, possess either MTA/*S*-adenosylhomocysteine (SAH) nucleosidase (MtnN) (Fig. 1; enzyme B) or MTA phosphorylase (MtnP) (Fig. 1; enzyme D) for the hydrolysis or phosphorolysis of MTA to 5-(methylthio)ribose or 5-(methylthio)ribose-1-P (P, phosphate), respectively (6, 11). Organisms such as Escherichia coli that live in sulfur-rich environments simply excrete 5-(methylthio)ribose (Fig. 1; compound 4) into the environment at the expense of organic sulfur (12). However, given that many organisms encounter sulfur-limiting environments, further recycling of MTA to regenerate a usable sulfur source, typically l-methionine, is necessary to maintain proper cellular sulfur pools. This is underpinned by the fact that nearly all eukaryotes possess the universal methionine salvage pathway (MSP), also referred to as the canonical MSP or, in plant systems, the Yang cycle (Fig. 1; black). The canonical pathway consists of six enzymatic steps: nucleosidase/phosphorylase (MtnP), isomerase (MtnA), dehydratase (MtnB), enolase/phosphatase (MtnC), dioxygenase (MtnD), and transaminase (MtnE) (Fig. 1; enzymes D to F and enzymes I to K) (13). This results in the conversion of MTA to adenine, formate, and l-methionine at the expense of inorganic phosphate, molecular oxygen, and a suitable amino acid as an amine donor. Moreover, this process is inherently aerobic due to the oxygen requirement of the dioxygenase.

While some bacteria also possess the canonical MSP, it is becoming increasingly evident that many bacteria have developed multiple variations of this pathway, some of which result in the production of novel metabolites, including isoprenoids and ethylene (14, 15). The *Bacillus* variation replaces the bifunctional enolase/phosphatase (MtnC concentration) (Fig. 1; enzyme I) with a RuBisCO-like protein (RLP), from the YkrW clade, functioning as a 2,3-diketo-5-methylthiopentyl-1-P enolase (MtnW) (Fig. 1; enzyme G), and a separate 2-hydroxy-3-keto-5-methylthiopent(1)ene-1-P phosphatase (MtnX) (Fig. 1; enzyme H) (16, 17). Similarly, the *Tetrahymena* variation replaces MtnB, MtnC, and MtnD with a single multifunctional fusion enzyme (MtnBD) catalyzing the dehydratase, enolase, dioxygenase, and, possibly, phosphatase reactions (18). In these cases, the products and oxygen requirements are the same as those of the canonical MSP.

Alternatively, some proteobacteria contain genes encoding the MTA-isoprenoid shunt MSP, originally discovered in Rhodospirillum rubrum (Fig. 1; blue) (14), which results in the formation of 1-deoxyxylulose-5-P (DXP) for isoprenoid metabolism and of volatile methanethiol (Fig. 1; compound 13) for l-methionine regeneration (14). Unlike the canonical MSP, the MTA-isoprenoid shunt is oxygen independent and functions under both aerobic and anaerobic growth conditions in R. rubrum (14, 19). Analogous to the canonical MSP, the first two enzymatic steps of the MTA-isoprenoid shunt are catalyzed by MTA phosphorylase (MtnP) and 5-(methylthio)ribose-1-P isomerase (MtnA) (Fig. 1; enzymes D and E). Next, an RLP from the DeepYkr clade catalyzes the 1,3-isomerization of the resultant 5-(methylthio)ribulose-1-P to 1-(methylthio)xylulose-5-P and 1-(methylthio)ribulose-5-P at a 3:1 ratio (Fig. 1; enzyme L). Subsequently a cupin-type 1-(methylthio)xylulose-5-P methylsulfurylase catalyzes the glutathione-mediated reduction of 1-(methylthio)xylulose-5-P to *S-*methylthio-glutathione and DXP (Fig. 1; enzyme M) (20, 21). During the process of glutathione regeneration, free methanethiol is proposed to be released either by the γ-glutamyl cycle, initiated by γ-glutamyl transferase (21), or by a putative specific methylthio-glutathione reductase (22). The liberated methanethiol couples to O-acetyl-l-homoserine (OAHS) catalyzed by the enzyme OAHS sulfhydrylase to regenerate l-methionine (Fig. 1; enzyme N) (14).

Further investigations into MTA metabolism in R. palustris and R. rubrum led to the discovery of an exclusively anaerobic ethylene-forming MSP (Fig. 1; orange) (15), which we formally name here the “DHAP-ethylene shunt” (for "dihydroxyacetone phosphate-methanethiol shunt") Like the canonical and MTA-isoprenoid shunt MSPs, the DHAP-ethylene shunt MSP proceeds via the activity of MtnP and MtnA (Fig. 1; enzymes D and E). However, the resultant 5-(methylthio)ribulose-1-P is then cleaved into dihydroxyacetone phosphate (DHAP) and 2-(methylthio)acetaldehyde by means of a class II aldolase-like protein (Ald2) functioning as a 5-(methylthio)ribulose-1P aldolase (Fig. 1; enzyme O). The 2-(methylthio)acetaldehyde is subsequently reduced by a putative alcohol dehydrogenase to 2-(methylthio)ethanol (Fig. 1; enzyme P), which is further metabolized by an unknown enzyme(s) to recycle the methylthio moiety, generating ethylene in the process.

Lastly, in R. rubrum, bona fide RuBisCO is also absolutely required to support anaerobic MTA metabolism. Complementation studies performed with mutant RuBisCO proteins indicate that RuBisCO may function differently from the typical ribulose-1,5-bisphosphate-dependent CO_2_ fixation reaction and is required for MTA metabolism. Complementation studies show also that all extant forms of RuBisCO (forms I, II, and III) can perform this unknown function, and metabolomics studies suggest that the unknown function may be linked to the formation of *S-*methyl-3-mercaptopyruvate and *S-*methyl-l-cysteine (23).

R. palustris is unique in that it possesses homologues for two RLPs (encoded by the *rlp1* and *rlp2* genes) and two RuBisCOs, (form I, encoded by *cbbLS*, and form II, encoded by *cbbM*) (24). RLP1 of R. palustris is homologous to the DeepYkr RLP from the R. rubrum MTA-isoprenoid shunt (Fig. 1; enzyme L), and RLP2 is homologous to the Chlorobaculum tepidum Photo RLP, which in C. tepidum is involved in a stress response and sulfur oxidation (25). Moreover, R. palustris contains all the requisite gene homologues for the MTA-isoprenoid shunt (Fig. 1; blue) (19) and also possesses a functional DHAP-ethylene shunt MSP (Fig. 1; orange) (15). As such, R. palustris is poised for metabolic versatility in its ability to metabolize MTA in order to maintain proper intracellular sulfur pools. However, the aerobic mechanisms of methionine salvage in this organism and, by extension, the function of the two distinct RLPs and RuBisCOs in sulfur metabolism are largely unknown.

In this study, we showed, through a combination of gene deletions and directed metabolite detection studies, that under aerobic conditions, R. palustris contains both a functional MTA-isoprenoid shunt and an additional aerobic MSP, which we name the “DHAP-methanethiol shunt MSP.” Analogous to the exclusively anaerobic DHAP-ethylene shunt from R. rubrum and R. palustris (Fig. 1; orange) (15), the R. palustris aerobic DHAP-methanethiol shunt initially proceeds by converting MTA to 2-(methylthio)ethanol and DHAP via MtnP, MtnA, Ald2, and a putative alcohol dehydrogenase (Fig. 1; enzymes D, E, O, and P). However, this novel R. palustris aerobic DHAP-methanethiol shunt MSP then employs an unknown enzyme(s) for methionine regeneration, resulting in metabolism of 2-(methylthio)ethanol into volatile methanethiol. This is in contrast to the results seen with the exclusively anaerobic DHAP-ethylene shunt MSP, which instead further metabolizes 2-(methylthio)ethanol via an unknown enzyme(s) into ethylene and an unknown organo-sulfur intermediate for methionine regeneration. Presumably, as with the R. rubrum MTA-isoprenoid shunt (Fig. 1; enzyme N) (14), the homologous R. palustris OAHS sulfhydrylase (Met17) regenerates methionine from the liberated methanethiol and O-acetyl-l-homoserine. Feeding experiments performed with ^14^C-labeled MTA indicated that the two pathways function simultaneously, suggesting a partitioning of MTA between DXP and DHAP for metabolic versatility. Furthermore, we demonstrate *in vivo* and *in vitro* that the R. palustris DeepYkr RLP enzyme (RLP1) catalyzes the 5-(methylthio)ribulose-1-P 1,3-isomerization reaction of the MTA-isoprenoid shunt to form 1-(methylthio)xylulose-5-P and 1-(methylthio)ribulose-5-P, whereas the Photo RLP enzyme (RLP2) does not. Finally, under aerobic conditions, both the MTA-isoprenoid shunt and DHAP-methanethiol shunt MSPs appear to be constitutively functional irrespective of the total available sulfur concentration to serve a general housekeeping function in converting MTA to methanethiol. Under anaerobic conditions, the DHAP-methanethiol shunt is inactive. These findings suggest a possible mechanism by which methanethiol, a key component of the global sulfur cycle, is biologically produced by bacteria.

## RESULTS

### R. palustris contains multiple aerobic methionine salvage pathways linked to methanethiol production.

**FIG 2:**
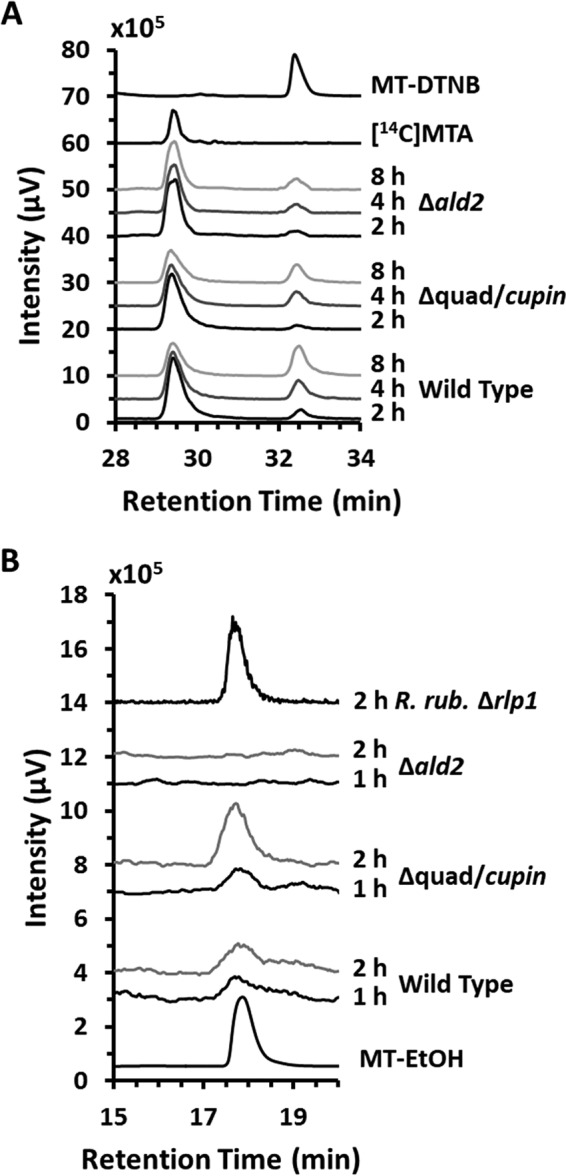
Identification of 2-(methylthio)ethanol and volatile methanethiol produced from [methyl-^14^C]MTA. (A) Methanethiol and (B) 2-(methylthio)ethanol were identified in various R. palustris stains fed aerobically with [methyl-^14^C]MTA for the indicated amounts of time (h) in the presence of DTNB to capture free thiols released from the cells. Metabolites were separated by reverse-phase HPLC with an in-line radiometric detector. Methanethiol as a DTNB adduct was the only free-thiol species observed. MT-DTNB, unlabeled methanethiol standard as a DTNB adduct detected by 320-nm-wavelength absorbance; [^14^C]MTA, [methyl-^14^C]MTA standard. An R. rubrum RLP deletion strain (*R. rub*. Δ*rlp1*) was fed anaerobically as a control for 2-(methylthio)ethanol identification since this strain was previously reported to produce 2-(methylthio)ethanol from MTA (15). MT-EtOH, unlabeled 2-(methylthio)ethanol standard detected by 215-nm-wavelength absorbance.

R. palustris strain CGA010 is able to grow aerobically on MTA as a sole sulfur source, indicating that it has a functional aerobic MSP for salvaging sulfur from MTA (Table 1) (Fig. S1). However, based on sequence homology, R. palustris does not possess any known homologous enzymes of the universal MSP (Fig. 1; black) save for MTA phosphorylase (*mtnP*, RPA4821), 5-(methylthio)ribose-1-P isomerase (*mtnA*, RPA4820), and a putative dioxygenase (*mtnD*, RPA2352). Rather, R. palustris encodes the following putative homologues to the R. rubrum MTA-isoprenoid shunt genes (Fig. 1; blue) (14, 15): 5-(methylthio)ribulose-1-P 1,3-isomerase (*rlp1*; RPA2169), 1-(methylthio)xylulose-5-P methylsulfurylase (*cupin*; RPA2170), and OAHS sulfhydrylase (*met17*; RPA2362 and RPA4251). In R. rubrum, aerobic and anaerobic metabolism of MTA leads to the formation of volatile methanethiol by virtue of the MTA-isoprenoid shunt MSP (Fig. 1; blue) (14, 23). Catalyzed by OAHS sulfhydrylase, methanethiol is coupled to O-acetyl-l-homoserine to regenerate l-methionine (Fig. 1; enzyme N); if not utilized, methanethiol is released into the extracellular environment (14). To determine if a similar MTA-isoprenoid shunt MSP occurred in R. palustris, cells were grown aerobically on MTA as the sole sulfur source, washed with sulfur-free media, and then fed [methyl-^14^C]MTA. Free thiols generated upon feeding were captured with Ellman’s reagent [5,5′-dithiobis-(2-nitrobenzoic acid) (DTNB)] and resolved by reverse-phase high-performance liquid chromatography (HPLC) with an in-line radiometric detector. As with R. rubrum (23), [methyl-^14^C]methanethiol was observed in R. palustris as a DTNB adduct (Fig. 2A), suggesting a functional MTA-isoprenoid shunt in R. palustris.

**TABLE 1:** R. palustris strains used in this study

R. palustris strain	Growth phenotype or description^a^	Reference	Aerobic growth doubling time (h)^b^	
			SO_4_^−2^	MTA
Wild type	CGA010, St^r^	43	22 ± 3	20 ± 3
Δ*rlp1*	Rlp1; DeepYkr clade RLP (ΔRPA2169)	15	25 ± 3	27 ± 3
Δ*rlp2*	Rlp2^—^; Photo clade RLP (ΔRPA0262)	15	27 ± 1	26 ± 1
Δ*rlp12*	Δ*rlp1* Δ*rlp2*	This study	27 ± 2	28 ± 3
Δ*cupin*	Cupin^—^; MTXu-5P methylsulfurylase (ΔRPA2170)	15	22 ± 3	27 ± 5
Δ*quad*	Δ*rlp1* Δ*rlp2* RbcL^—^ (ΔRPA1559) CbbM^—^ (ΔRPA4041)	This study	19 ± 2	19 ± 3
Δ*quad* Δ*cupin*	Δ*rlp1* Δ*rlp2* Δ*rbcL* Δ*cbbM* Δcupin	This study	20 ± 3	22 ± 2
Δ*mtnP*	MtnP^—^; MTA phosphorylase (ΔRPA4821)	15	19 ± 2	NG
Δ*mtnP*::pBBR1	Δ*mtnP* complemented with pBBR1	15	19 ± 2	NG
Δ*mtnP*::pBBR1-RpMtnP	Δ*mtnP* complemented with pBBR1 bearing *mtnP*	15	20 ± 3	20 ± 2
Δ*mtnA*	ΔMTR-1P isomerase (RPA4820)	15	21 ± 2	NG
Δ*mtnA*::pBBR1	Δ*mtnA* complemented with pBBR1	15	25 ± 1	NG
Δ*mtnA*::pBBR1-RpMtnA	Δ*mtnA* complemented with pBBR1 bearing *mtnA*	15	23 ± 2	18 ± 2
Δ*ald2*	Ald2^—^; MTRu-1P aldolase (ΔRPA4655)	15	19 ± 3	21 ± 2
Δ*rlp1* Δ*ald2*	Δ*rlp1* Δ*ald2*	This study	20 ± 6	60 ± 3
Δ*rlp1* Δ*ald2*::pBBR1	Δ*rlp1* Δ*ald2* complemented with pBBR1	This study	19 ± 1	70 ± 5
Δ*rlp1* Δ*ald2*::pBBR1-RpAld2	Δ*rlp1* Δ*ald2* complemented with pBBR1 bearing *ald2*	This study	21 ± 3	17 ± 3
Δ*rlp2* Δ*ald2*	Δ*rlp2* Δ*ald2*	This study	19 ± 4	17 ± 4
Δ*rlp12* Δ*ald2*	Δ*rlp1* Δ*rlp2* Δ*ald2*	This study	21 ± 3	57 ± 4
Δ*rlp12* Δ*ald2*::pBBR1	Δ*rlp12* Δ*ald2* complemented with pBBR1	This study	17 ± 1	65 ± 2
Δ*rlp12* Δ*ald2*::pBBR1-RpAld2	Δ*rlp12* Δ*ald2* complemented with pBBR1 bearing *ald2*	This study	21 ± 1	18 ± 3

a R. palustris strain CGA009 gene locus identification (ID) numbers from NCBI given in parentheses. St^r^, streptomycin resistance.

b Doubling times were calculated by least-squares fit of the growth data (during the period in which the growth was in the exponential phase; see Fig. S1 to S6) to the model ln(growth) = *kt* + *b* such that doubling time = ln(2)/*k*. Error bars represent standard errors of the fit data. NG, no growth observed.

10.1128/mBio.00407-18.1 FIG S1  Aerobic growth of the indicated R. palustris strains by the use of 500 μM sulfate (circles), 500 μM MTA (squares), or no sulfur (triangles) as the sole sulfur source. Download FIG S1, TIF file, 0.1 MB. Copyright © 2018 Miller et al. 2018 Miller et al. This content is distributed under the terms of the Creative Commons Attribution 4.0 International license.

Distinguishing enzymes of the MTA-isoprenoid shunt in R. rubrum are the DeepYkr RLP and cupin-type methylsulfurylase (Fig. 1; enzymes L and M) (14). Sequence homology and gene organization suggested that the R. palustris DeepYkr RLP (RLP1) (53% identity; E value 9e^−121^) and cupin (56% identity; E value 3e^−50^) homologues may function like their R. rubrum counterparts. Additionally, previous *in vitro* work on functional diversity in the RLP enzyme family indicated that the R. palustris Photo RLP (RLP2) may also catalyze an isomerization reaction similar to that seen with the bona fide R. rubrum DeepYkr RLP (22). Therefore, we initially inactivated the DeepYkr RLP (Δ*rlp1*) and Photo RLP (Δ*rlp2*) genes in R. palustris. Mutant strains lacking a functional *rlp1* gene or *rlp2* gene or both showed growth similar to that shown by the wild type under aerobic conditions using MTA as the sole sulfur source (Table 1) (Fig. S1).

**FIG 3:**
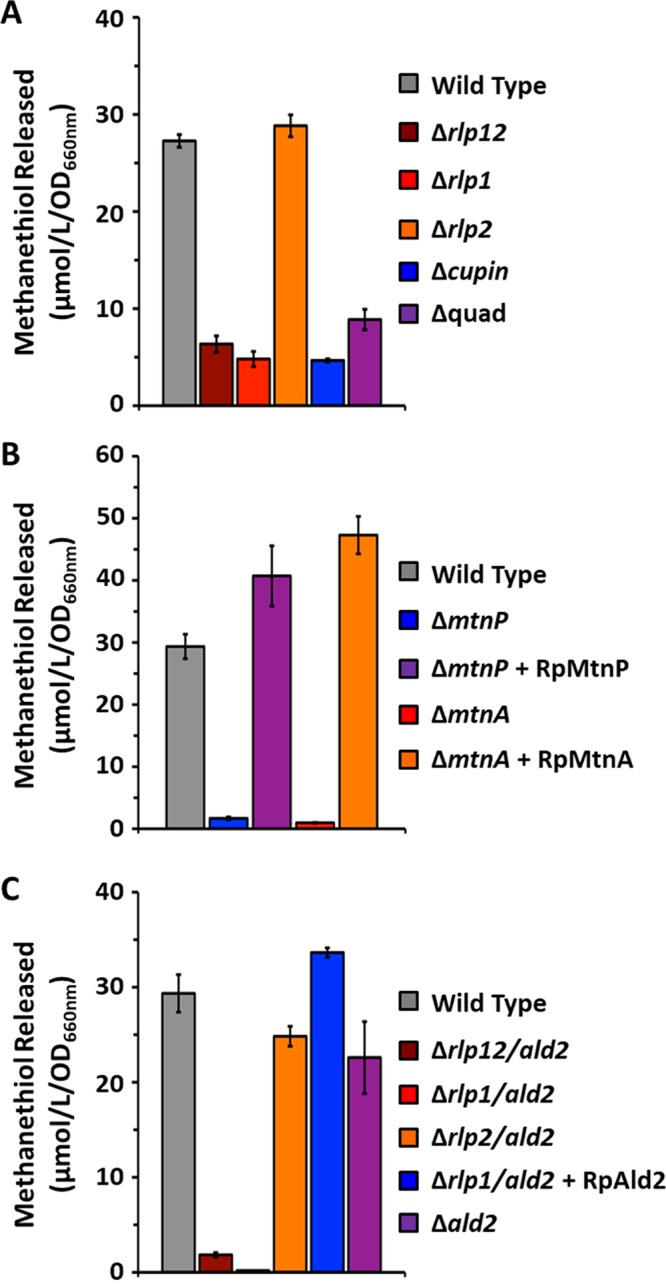
Quantification of volatile methanethiol released by R. palustris. (A) Cultures of each indicated strain were initially grown aerobically in the presence of MTA, washed in sulfur-free media containing DTNB, and then fed MTA for 12 h. (B and C) Cultures were initially grown aerobically in the presence of sulfate, washed in sulfur-free media containing DTNB and then fed for 12 h with MTA. The Δ*mtnP* and Δ*mtnA* deletion strains (B) and the Δ*rlp12*, Δ*rlp1*, Δ*rlp2*, and Δ*ald2* deletion strains (C) were complemented with pBBR1-RpMtnP (+ RpMtnP; MTA phosphorylase), pBBR1-RpMtnA [+ RpMtnA; 5-(methylthio)ribose-1-P isomerase], and pBBR1-RpAld2 [+ RpAld2; 5-(methylthio)ribulose-1-P aldolase]. Volatile methanethiol released by the cells was captured as a DTNB adduct and quantified by reverse-phase HPLC based on a standard calibration curve. Data corresponding to the total methanethiol released by the cells during feeding are given in micromoles detected per liter of culture per culture optical density measured at 660 nm (μmol/liter/OD_660_). Error bars indicate standard deviations of results from *n* = 3 independent feeding experiments.

To determine if methanethiol was still being produced in the absence of a functional RLP in R. palustris, cells were grown aerobically on MTA as the sole sulfur source, washed with sulfur-free media, and then fed with MTA. Methanethiol released by the cell upon feeding with MTA was captured with DTNB and measured by reverse-phase chromatography. In the absence of a functional RLP1 (Δ*rlp1* strain or Δ*rlp12* strain), methanethiol was still produced, but at levels that were 4-to-5-fold lower than the wild-type level (Fig. 3A). The level of methanethiol liberated by the R. palustris Δ*rlp2* strain was similar to the wild-type level. This indicated that the R. palustris DeepYkr RLP (RLP1) and not the Photo RLP (RLP2) was likely involved in the putative MTA-isoprenoid shunt. To further determine the functionality of the putative MTA-isoprenoid shunt, the homologous cupin gene was inactivated in the R. palustris wild-type strain (Δ*cupin*). Again, growth on MTA as the sole sulfur source was unaffected (Table 1) (Fig. S1), but the same 4-to-5-fold decrease in the methanethiol level was observed (Fig. 3A). These results indicated that R. palustris possesses a putative MTA-isoprenoid shunt in which the DeepYkr RLP and cupin participate. Moreover, since aerobic growth on MTA occurred in the absence of RLP or cupin and since methanethiol was still produced, a separate aerobic MSP must be functional in R. palustris that is also linked to methanethiol production. Therefore, we sought to verify the MTA-isoprenoid shunt as well as to characterize this putative additional aerobic MSP in R. palustris.

### Neither of the RuBisCOs of R. palustris contributes to aerobic MTA metabolism.

Previous studies of R. rubrum revealed that a bona fide RuBisCO is absolutely required to support anaerobic growth on MTA as the sole sulfur source (26). This requirement does not appear to be true simply for cycling the RuBisCO substrate, ribulose-1,5-bisphoshate, because certain mutant RuBisCO enzymes severely compromised in carbon fixation activity are still able to support MTA-dependent growth (23). Rather, it appears that RuBisCO functions specifically in MTA metabolism in R. rubrum via an unknown mechanism (26). Recent studies have demonstrated that in both R. palustris and R. rubrum, RuBisCO not only is synthesized anaerobically but also may be synthesized under aerobic or microaerophilic conditions (27, 28). Indeed, under aerobic growth conditions, deletion of RuBisCO in R. rubrum (strain I19* [23]) resulted in a delayed growth phenotype aerobically (Fig. S2) when MTA was used as the sole sulfur source compared to sulfate, suggesting a possible role in aerobic MTA metabolism as well. Therefore, we considered the possibility that one or both of the R. palustris RuBisCOs might be functioning in aerobic MTA metabolism. Both the form I RuBisCO gene (*cbbLS*) and the form II RuBisCO gene (*cbbM*), in conjunction with the *cupin* gene, were deleted in the Δ*rlp12* background to form strain Δ*quad* Δ*cupin*. Inactivation of the RuBisCOs had no effect on either aerobic or anaerobic growth using MTA versus sulfate as the sole sulfur source (Table 1) (Fig. S2 and S3). The levels of methanethiol production by R. palustris showed negligible differences between the Δ*quad* Δ*cupin* and Δ*rlp12* strains [*t* = 3.23; *P* value = 0.05; confidence interval (CI) = 0.05] (Fig. 3A), further indicating that RuBisCO was not involved in methanethiol metabolism from MTA. Together, these results support the conclusion that neither of the two RuBisCOs functions in MTA metabolism in R. palustris.

10.1128/mBio.00407-18.2 FIG S2  Aerobic growth of the indicated R. palustris (A and B) and R. rubrum (C and D) strains by the use of 500 μM sulfate (circles), 500 μM MTA (squares), or no sulfur (triangles) as the sole sulfur source. Download FIG S2, TIF file, 0.1 MB. Copyright © 2018 Miller et al. 2018 Miller et al. This content is distributed under the terms of the Creative Commons Attribution 4.0 International license.

10.1128/mBio.00407-18.3 FIG S3  Anaerobic growth of the indicated R. palustris (A and B) strains by the use of 500 μM sulfate (circles), 500 μM MTA (squares), or no sulfur (triangles) as the sole sulfur source. Download FIG S3, TIF file, 0.2 MB. Copyright © 2018 Miller et al. 2018 Miller et al. This content is distributed under the terms of the Creative Commons Attribution 4.0 International license.

### MtnP, MtnA, and Ald2 function in the additional R. palustris aerobic MSP.

Our previous studies of R. rubrum and R. palustris anaerobic MTA metabolism established the strictly anaerobic DHAP-ethylene shunt MSP (15). Here, MTA phosphorylase (MtnP), 5-(methylthio)ribose-1-P isomerase (MtnA), a class II aldolase-like protein (Ald2) functioning as a 5-(methylthio)ribulose-1P aldolase, and a putative alcohol dehydrogenase (Fig. 1; enzymes D and E and enzymes O and P) sequentially enabled the metabolism of MTA to 2-(methylthio)ethanol and DHAP. Subsequently, but only under anaerobic growth conditions, the 2-(methylthio)ethanol was further metabolized to ethylene and an unknown organo-sulfur intermediate for sulfur salvage (15). Interestingly, R. palustris could also grow aerobically on 2-(methylthio)ethanol as a sole sulfur source, suggesting that MtnP, MtnA, and Ald2 may function in this organism in an aerobic MSP involving 2-(methylthio)ethanol that is different from the exclusively anaerobic DHAP-ethylene shunt MSP. Therefore, we systematically quantified the growth of and methanethiol production from R. palustris MTA phosphorylase (Δ*mtnP*), 5-(methylthio)ribose-1P isomerase (Δ*mtnA*), and aldolase (Δ*ald2*) gene deletion strains (Table 1).

As observed with R. rubrum (14), inactivation of MtnP and MtnA resulted in compromised aerobic growth on MTA as the sole sulfur source (Table 1) (Fig. S4). Furthermore, MTA feeding experiments performed with *ΔmtnP* and *ΔmtnA* deletion strains initially grown on sulfate showed no methanethiol production, in contrast to the wild-type results (Fig. 3B). Upon complementing the Δ*mtnP* and Δ*mtnA* deletion strains with their respective genes expressed in *trans* from a plasmid, both growth (Table 1) (Fig. S4) and methanethiol production (Fig. 3B) were restored to wild-type levels. This demonstrated that MTA phosphorylase (MtnP) and 5-(methylthio)ribose-1-P isomerase (MtnA) are the first two requisite enzymes for both aerobic MSPs, the putative MTA-isoprenoid shunt and the additional pathway, in R. palustris.

10.1128/mBio.00407-18.4 FIG S4  Aerobic growth of the indicated R. palustris strains by the use of 500 μM sulfate (circles), 500 μM MTA (squares), or no sulfur (triangles) as the sole sulfur source. (A to C) An MTA phosphorylase deletion strain (Δ*mtnP*) was complemented with pBBR1 broad-host-range plasmid bearing the R. palustris *mtnP* gene inserted into the multicloning site (B) and with empty pBBR1 plasmid (C). (D to F) A 5-(methylthio)ribose-1-P isomerase deletion strain (Δ*mtnA*) (D) was complemented with pBBR1 broad-host-range plasmid bearing the R. palustris *mtnA* gene inserted into the multicloning site (E) and with empty pBBR1 plasmid (F). Download FIG S4, TIF file, 0.1 MB. Copyright © 2018 Miller et al. 2018 Miller et al. This content is distributed under the terms of the Creative Commons Attribution 4.0 International license.

**FIG 4:**
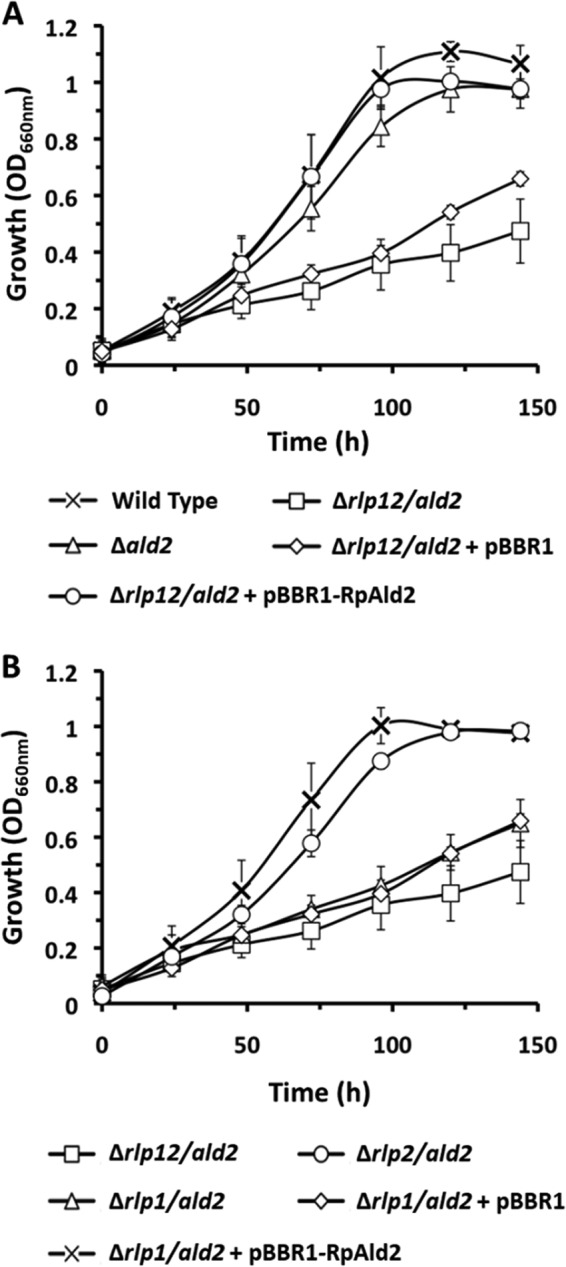
Aerobic growth of R. palustris strains by the use of 500 μM MTA as the sole sulfur source. Culture optical density was measured at a 660-nm wavelength (OD_660_). Error bars indicate standard deviations of results from *n* = 3 independent growth experiments. Panels A and B represent the same experiment, but separated into two panels for ease of reading/viewing.

Next, the putative 2-(methylthio)ribulose-1-P aldolase (*ald2*) gene was inactivated in both the wild-type strain and the Δ*rlp12* deletion strain to construct strains Δ*ald2* and Δ*rlp12* Δ*ald2*, respectively (Table 1). While the aldolase deletion in the wild-type background (*Δald2*) was still capable of aerobic growth on MTA as the sole sulfur source, the Δ*rlp12* Δ*ald2* strain exhibited very poor growth (Table 1) (Fig. 4A and S5). Moreover, while the *Δald2* deletion strain could still produce methanethiol at ~75% of the wild-type level, negligible methanethiol was observed in the Δ*rlp12* Δ*ald2* deletion strain (Fig. 3C). Complementation of the Δ*rlp12* Δ*ald2* strain with the aldolase expressed in *trans* not only restored growth (Table 1) (Fig. S4A and S5) but also restored methanethiol production to nearly wild-type levels (Fig. 3C). These results confirmed that R. palustris has two primary MSPs, a putative MTA-isoprenoid shunt and a second aerobic MSP utilizing Ald2. Again, it was apparent that both pathways required MTA phosphorylase (MtnP) and 5-(methylthio)ribose-1-P isomerase (MtnA) for the initial metabolism of MTA, and both pathways resulted in the generation of volatile methanethiol.

10.1128/mBio.00407-18.5 FIG S5  Aerobic growth of the indicated R. palustris strains by the use of 500 μM sulfate (circles), 500 μM MTA (squares), or no sulfur (triangles) as the sole sulfur source. (A) 5-(Methylthio)ribulose-1-P aldolase deletion strain (Δ*ald2*). (B to D) A RLP1/RLP2/5-(methylthio)ribulose-1-P aldolase deletion strain (Δ*rlp12 ald2*) (B) was complemented with a pBBR1 broad-host-range plasmid bearing the R. palustris *ald2* gene inserted into the multicloning site (C) and with empty pBBR1 plasmid (D). Download FIG S5, TIF file, 0.1 MB. Copyright © 2018 Miller et al. 2018 Miller et al. This content is distributed under the terms of the Creative Commons Attribution 4.0 International license.

### Ald2 links MTA metabolism to 2-(methylthio)ethanol as part of an aerobic methanethiol-producing MSP.

We next sought to identify the *in vivo* product(s) of the Ald2-catalyzed reaction of the additional methanethiol-producing MSP. Under anaerobic conditions, in both R. palustris and R. rubrum, Ald2 functions as a 5-(methylthio)ribulose-1-P aldolase to form DHAP and 2-(methylthio)acetaldehyde as part of the DHAP-ethylene shunt MSP (14, 15). To determine if a similar sequence was occurring aerobically *in vivo*, the wild-type, Δ*quad* Δ*cupin*, and Δa*ld2* strains were fed with [methyl-^14^C]MTA. Metabolites were extracted and resolved by reverse-phase HPLC with an in-line radiometric detector. As observed for anaerobic MTA metabolism (15), 2-(methylthio)ethanol was produced in the wild-type strain and was undetectable in the aldolase deletion strain (Fig. 2B). In addition, 2-(methylthio)ethanol was enhanced in the Δ*quad* Δ*cupin* strain in which the MTA-isoprenoid shunt was inactivated via knockouts of RLP1 and cupin (*vide infra*). As previously reported (15), observance of 2-(methylthio)ethanol and not 2-(methylthio)acetaldehyde *in vivo* is likely due to rapid conversion of the aldehyde to the less reactive alcohol moiety, presumably by an alcohol dehydrogenase. As such, these results indicate that Ald2 also functions *in vivo* as a 5-(methylthio)ribulose-1-P aldolase to form DHAP and 2-(methylthio)acetaldehyde under aerobic growth conditions in R. palustris.

Methanethiol captured by DTNB after feeding the Δ*quad* Δ*cupin* strain with [methyl-^14^C]MTA was observed as [methyl-^14^C]methanethiol (Fig. 2A), confirming that the methanethiol produced by the second MSP in which Ald2 participates was also derived from MTA, presumably via 2-(methylthio)ethanol as an intermediate. This was further supported by the observation that R. palustris strains fed aerobically with 2-(methylthio)ethanol produced methanethiol at a rate similar to that seen when they were fed with MTA (Table 2). These observations are consistent with the conclusion that R. palustris possesses an aerobic DHAP-methanethiol shunt MSP analogous to the anaerobic DHAP-ethylene shunt MSP. There, DHAP and 2-(methylthio)acetaldehyde are produced from MTA by the sequential action of MtnP, MtnA, and Ald2 (14, 15). It is likely an alcohol dehydrogenase catalyzes reduction of 2-(methylthio)acetaldehyde to the observed 2-(methylthio)ethanol, which is further metabolized via some unknown enzyme(s) to produce methanethiol for sulfur salvage (Fig. 6; green).

**TABLE 2:** Rate of methanethiol release from R. palustris

Strain	Growth	[SO_4_^−2^] (μM)	[MTA] (μM)	[MT-EtOH] (μM)	Rate of MT release (μmol/liter/h/OD)^a^
Wild type	Aerobic	0	1,000	0	3.89 ± 0.19
		0	0	1,000	1.72 ± 0.56
	Anaerobic	0	1,000	0	3.97 ± 1.07
		0	0	1,000	n.d.
Δ*rlp1*	Aerobic	0	1,000	0	0.73 ± 0.27
		0	0	1,000	1.66 ± 0.44
	Anaerobic	0	1,000	0	n.d.
		0	0	1,000	n.d.
					
Wild type	Aerobic	0	0	0	n.d.
		10	0	0	0.01 ± 0.02
		30	0	0	0.02 ± 0.01
		100	0	0	0.15 ± 0.01
		300	0	0	0.15 ± 0.02
		1,000	0	0	0.14 ± 0.02
					
Wild type	Aerobic	10	1,000	0	3.87 ± 1.19
		100	1,000	0	1.91 ± 0.37
		1,000	1,000	0	2.56 ± 0.97

a Data represent the rate of methanethiol release from cells in micromoles of methanethiol released per liter of culture per hour per cell culture optical density measured at a 660-nm wavelength (μmol/liter/h/OD). Methanethiol was measured as a DTNB adduct by HPLC based on a standard calibration curve. Error bars indicate standard deviations of results from *n* = 3 independent cultures. n.d., no methanethiol detected.

The [methyl-^14^C]MTA feeding experiments using the wild-type strain versus strains in which the MTA-isoprenoid shunt (strain *Δquad* Δ*cupin*) or DHAP-methanethiol shunt (strain *Δald2*) was inactivated enabled measurement of an approximate mass balance in the conversion of MTA to methanethiol for each pathway. Upon feeding each strain, there was a linear decrease in the level of [methyl-^14^C]MTA due to consumption by the cells and a concomitant linear increase in the level of volatile [methyl-^14^C]methanethiol released, which was captured and detected as a DNTB adduct. Given that a fraction of the methanethiol formed would have been oxidized or reincorporated into the cell, presumably as methionine and subsequent metabolites, before it could have been captured, the ratio of the rate of methanethiol captured to the rate of MTA consumed gives a lower limit on the mass balance. For the wild-type strain, 62% ± 10% of the MTA consumed was captured as methanethiol on a molar basis. The pathways appeared to contribute similarly to this process. For the DHAP-methanethiol shunt (strain Δ*quad* Δ*cupin*), 41% ± 6% of the MTA consumed was captured as methanethiol, and for the MTA-isoprenoid shunt (strain *Δald2*), it was 32% ± 4%. Therefore, both the MTA-isoprenoid shunt and the MTA-DHAP shunt appreciably contribute to the aerobic conversion of MTA into methanethiol in R. palustris.

### Directed metabolite analysis confirms the presence of the MTA-isoprenoid shunt and the role of RLP1 in R. palustris.

Disruption of the second MSP via inactivation of the *ald2* gene permitted detailed characterization of the putative MTA-isoprenoid shunt, in particular, the possible role of the two RLPs in this pathway. For these studies, [methyl-^14^C]MTA was fed to the Δ*ald2* strain in which both RLPs were active. Free thiols were captured by DTNB and subsequently resolved by HPLC, revealing the production of [methyl-^14^C]methanethiol (Fig. 2A). This, coupled with the fact that the Δ*rlp12* Δ*ald2* strain produced negligible amounts of methanethiol, indicated that in the absence of a functional *ald2* gene, the putative MTA-isoprenoid shunt is the likely source for methanethiol. To determine whether RLP1 or RLP2 (or both) functions in this pathway, we separately inactivated each gene in the Δ*ald2* background to form strain Δ*rlp1* Δ*ald2* or strain Δ*rlp2* Δ*ald2*, respectively (Table 1). The Δ*rlp1* Δ*ald2* strain exhibited the same poor growth phenotype as the Δ*rlp12* Δ*ald2* strain, whereas the Δ*rlp2* Δ*ald2* strain grew similarly to the Δ*ald2* strain on MTA as the sole sulfur source (Table 1) (Fig. 4B and S6). In addition, upon being fed with MTA, the Δ*rlp1* Δ*ald2* strain produced negligible amounts of methanethiol, whereas the Δ*rlp2* Δ*ald2* strain produced methanethiol at levels similar to that produced by the Δ*ald2* strain (Fig. 3C). Together with the initial knockout studies in the wild-type background, these results establish that RLP1 functions in MTA metabolism, whereas RLP2 and the two RuBisCOs do not.

10.1128/mBio.00407-18.6 FIG S6  Aerobic growth of the indicated R. palustris strains by the use of 500 μM sulfate (circles), 500 μM MTA (squares), or no sulfur (triangles) as the sole sulfur source. (A to C) A RLP1/5-(methylthio)ribulose-1-P aldolase deletion strain (Δ*rlp1 ald2*) (A) was complemented with a pBBR1 broad-host-range plasmid bearing the R. palustris *ald2* gene inserted into the multicloning site (B) and with empty pBBR1 plasmid (C). (D) RLP2/5-(methylthio)ribulose-1-P aldolase deletion strain (*Δrlp2 ald2*). Download FIG S6, TIF file, 0.1 MB. Copyright © 2018 Miller et al. 2018 Miller et al. This content is distributed under the terms of the Creative Commons Attribution 4.0 International license.

**FIG 5:**
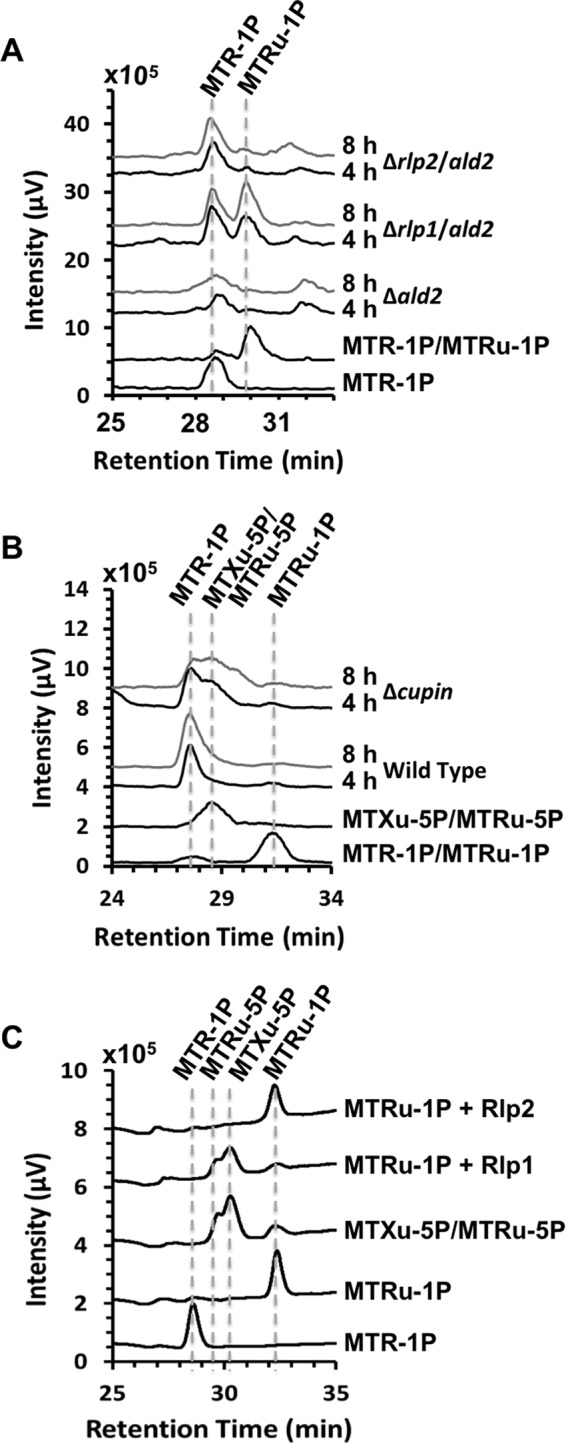
Identification of MTA-isoprenoid shunt MSP intermediates. (A and B) *In vivo* identification of 5-(methylthio)ribose-1-P (MTR-1P), 5-(methylthio)ribulose-1-P (MTRu-1P), and 1-(methylthio)xylulose-5-P (MTXu-5P). Cultures of each indicated strain were initially grown aerobically in the presence of MTA, washed in sulfur-free media, and then fed for the indicated amount of time (indicated in hours) with [methyl-^14^C]MTA. (A) Metabolites were extracted and ^14^C-labeled intermediates resolved in by HILIC as reported previously (14) and identified by inline radiometric detector based on known standards. (B) Chromatography conditions were optimized to enhance separation of the species (see Materials and Methods for details). (C) *In vitro* verification of R. palustris RLP1 activity corresponding to an MTRu-1P 1,3-isomerase based on known standards. Metabolites were detected by UV absorbance at a 215-nm wavelength (and by use of an in-line radiometric detector; see Fig. S8A).

In order to identify the specific *in vivo* RLP reaction, strains Δ*ald2*, Δ*rlp1* Δ*ald2*, and Δ*rlp2* Δ*ald2* were fed [methyl-^14^C]MTA. Generated ^14^C-labeled metabolites were extracted and resolved by the use of a hydrophilic interaction liquid chromatography (HILIC) HPLC instrument equipped with an in-line radiometric detector. Metabolites were identified based on known standards (Fig. 5A). In all 3 strains, 5-(methylthio)ribose-1-P and 5-(methylthio)ribulose-1-P, which are produced by the MTA phosphorylase (MtnP) and 5-(methylthio)ribose-1-P isomerase (MtnA) enzyme reactions, respectively, were identified (Fig. 1; enzymes D and E) (14, 19). Moreover, while 5-(methylthio)ribulose-1-P was observed at low levels in the Δ*ald2* and Δ*rlp2* Δ*ald2* strains, this compound was observed to have built up in the *Δrlp1* Δ*ald2* strain (Fig. 5A), further supporting the conclusion that RLP1 functions as the requisite 5-(methylthio)ribulose-1-P 1,3-isomerase in the MTA-isoprenoid shunt.

**FIG 6:**
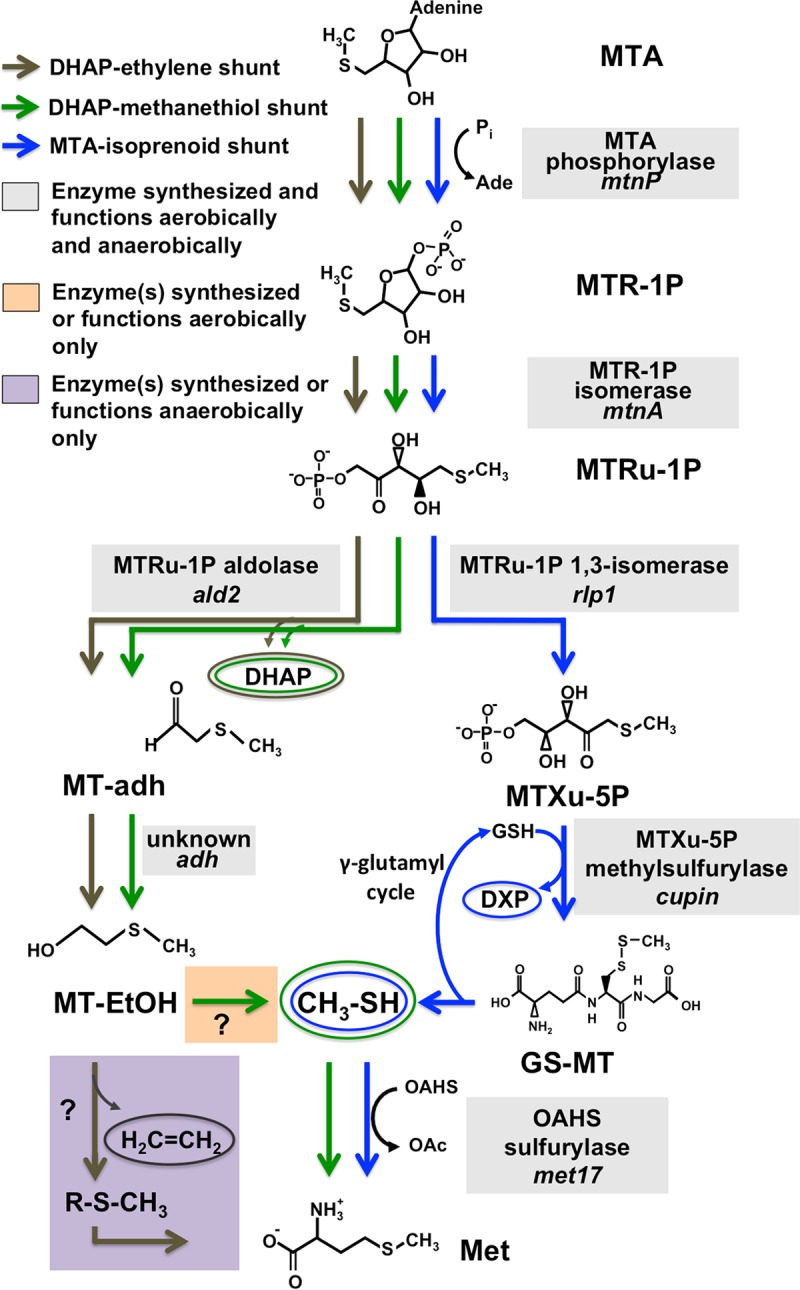
MTA metabolism in R. palustris. (Blue) Oxygen-independent MTA-isoprenoid shunt MSP, which functions aerobically (this study) and likely anaerobically (15). (Green) Aerobic DHAP-methanethiol shunt MSP (this study). (Gray) Anaerobic DHAP-ethylene shunt MSP (15). Note that it is likely the same putative unknown alcohol dehydrogenase (*adh*) which functions both aerobically in the MTA-methanethiol shunt and anaerobically in the MTA-ethylene shunt MSPs. However, this step could be catalyzed by different enzymes in the two different scenarios. Compounds were as follows: 5-(methylthio)ribose-1-P (MTR-1P), 5-(methylthio)ribulose-1-P (MTRu-1P), 1-(methylthio)xylulose-5-P (MTXu-5P), 2-(methylthio)acetaldehyde (MT-adh), 2-(methylthio)ethanol (MT-EtOH), 1-deoxyxylulose-5-P (DXP), glutathione (GSH), *S-*methylthio-glutathione (GS-MT), O-acetyl-l-homoserine (OAHS), acetate (OAc). γ-Glutamyl cycle, metabolism of glutathione adducts is initiated by γ-glutamyl transferase, resulting in recovery of glutamate, glycine, and cysteine, which are utilized to regenerate glutathione; the product has been observed to be constitutively expressed in R. rubrum, and the pathway is a proposed route for methanethiol release from *S-*methylthio-glutathione (21).

The inability to observe any specific RLP reaction products in these strains was likely due to the kinetic properties of R. palustris MtnA versus successive steps. In R. rubrum, the catalytic rate (*k*_cat_) of MtnA was low compared to those seen with other enzymes in the pathway. As a result, 5-(methylthio)ribulose-1-P was the dominant species observed and 1-(methylthio)xylulose-5-P was not observable unless the cupin-type methylsulfurylase was inactivated to prevent further metabolism (14). Predicting a similar trend in R. palustris, we therefore fed the R. palustris wild-type and Δ*cupin* strains with [methyl-^14^C]MTA (Fig. 5B). In the absence of a functional cupin, 1-(methylthio)xylulose-5-P was observed to build up in the cells. Taken together, these results were consistent with the conclusion that *in vivo* RLP1 but not RLP2 functioned as a 5-(methylthio)ribulose-1-P 1,3-isomerase and that the cupin functioned as a 1-(methylthio)xylulose-5-P methylsulfurylase as part of a functional MTA-isoprenoid shunt (Fig. 6; blue).

As an additional verification that RLP1 and not RLP2 was performing the 5-(methylthio)ribulose-1-P 1,3-isomerization reaction, we recombinantly expressed and purified the R. rubrum *and* R. palustris RLPs by His_6_-affinity nickel chromatography (see Fig. S7 in the supplemental material). Each RLP was observed as a dimer based on size exclusion chromatography elution times compared to molecular weight standards, consistent with previous reports for the R. palustris RLP2 and other RLPs (29). Each RLP was supplied with [methyl-^14^C]5-(methylthio)ribulose-1-P as a substrate, and products were resolved by HILIC HPLC or reverse-phase HPLC as described above. As in the case of the R. rubrum RLP, the R. palustris RLP1 also catalyzed the conversion of 5-(methylthio)ribulose-1-P to 1-(methylthio)xylulose-5-P and 1-(methylthio)ribulose-5-P at a 3:1 ratio (Fig. 5C; see also Fig. S8A). In contrast, any products formed from 5-(methylthio)ribulose-1-P by the mediation of RLP2 were below detectable limits and most of the substrate remained unreacted as previously reported (22). Specific formation of 1-(methylthio)xylulose-5-P was further verified by coupled assay with the R. rubrum cupin to form methanethiol (Fig. S8B). Subsequent kinetic rate measurement of product formation by the RLP from R. rubrum gave a *k*_cat_ value of 1.2 ± 0.1 s^−1^; the R. palustris RLP1 had a *k*_cat_ value of 1.3 ± 0.1 s^−1^, while R. palustris RLP2 showed no detectable activity with 5-(methylthio)ribulose-1-P as the substrate. These results confirmed that RLP1 primarily functions as the 5-(methylthio)ribulose-1-P 1,3-isomerase in the R. palustris MTA-isoprenoid shunt.

10.1128/mBio.00407-18.7 FIG S7  Recombinant proteins used in this study. SDS polyacrylamide gel electrophoresis of heterologously expressed and purified proteins was used for *in vitro* characterization. Lane 1, B. subtilis MTR kinase; lane 2, B. subtilis MTR-1-P isomerase; lane 3, R. rubrum RLP; lane 4, R. palustris RLP1; lane 5, R. palustris RLP2; lane 6, E. coli DXP reductoisomerase; lane 7, R. rubrum 1-(methylthio)xylulose-5-P methylsulfurylase (cupin). Download FIG S7, TIF file, 0.6 MB. Copyright © 2018 Miller et al. 2018 Miller et al. This content is distributed under the terms of the Creative Commons Attribution 4.0 International license.

10.1128/mBio.00407-18.8 FIG S8  Specific formation of 1-(methylthio)xylulose-5-P by R. palustris DeepYkr RLP. (A) Specific products of RLP1 and RLP2 enzymatic reactions utilizing [methyl-^14^C]5-(methylthio)ribulose-1-P (MTRu-1P) as the substrate from the experiment described in the Fig. 5C legend were also detected by subsequent use of an in-line radiometric detector. Due to mixing of HPLC effluent with scintillation fluid in the radiation detection flow cell, the resulting broadening of the peaks prevented resolution of 1-(methylthio)xylulose-5-P (MTXu-5-P) and 1-(methylthio)ribulose-5-P compared to detection by the use of a UV absorbance flow cell, which could separate the two species due to minimal broadening of the peaks (see Fig. 5C). (B) To further verify specific production of 1-(methylthio)xylulose-5-P, the reaction mixtures described in the panel A legend were further incubated in the presence of R. rubrum 1-(methylthio)xylulose-5-P methysylfhydrylase (cupin) and quenched with DTNB to capture any free thiols produced. [Methyl-^14^C]methanethiol was identified as a DTNB adduct (MT-DTNB) by reverse-phase HPLC performed with an in-line radiometric detector. Download FIG S8, TIF file, 0.3 MB. Copyright © 2018 Miller et al. 2018 Miller et al. This content is distributed under the terms of the Creative Commons Attribution 4.0 International license.

### MTA metabolism for volatile methanethiol production appears to be a general housekeeping function.

Under sulfur-limiting conditions, various MSPs are observed to be regulated, including the canonical MSP in *Bacillus* sp. (30, 31) and the anaerobic DHAP-ethylene shunt MSP of R. rubrum and R. palustris (15). For the R. rubrum MTA-isoprenoid shunt, previous transcriptomics and proteomics studies showed that enzymes MtnP, MtnA, RLP, and cupin (Fig. 1; enzymes D and E and enzymes L and M) were negligibly regulated whereas the O-acetyl-l-homoserine sulfhydrylase (Fig. 1; enzyme N) was highly upregulated when MTA was used as the sole sulfur source (14, 19, 21). These results were consistent with the observation that cells growing on sulfate that were then fed with MTA produced methanethiol at the same rate as cells grown on MTA and then fed with MTA (14). In other words, the methanethiol-forming sequence appeared to have a general housekeeping function for metabolizing MTA to methanethiol. Then, the O-acetyl-l-homoserine sulfhydrylase was regulated to generate l-methionine when required. However, those previous studies did not address the issue of whether the presence of environmentally available sulfate regulated the formation of methanethiol from MTA.

Given that both R. palustris aerobic MSPs led to volatile methanethiol production, we sought to determine if MTA metabolism occurred aerobically in the presence of sulfate by detecting methanethiol release from wild-type cells fed with various combinations of sulfate and MTA. The rate of liberation of methanethiol from fed cells was measured by methanethiol capture with DTNB and detection by reverse-phase HPLC as described above (Table 2). Regardless of the sulfate concentration, volatile methanethiol was generated from the supplied MTA (1 mM) at the same rate. These observations suggest that, unlike the ethylene-forming step of the anaerobic DHAP-ethylene shunt (15), the presence of sulfate does not regulate aerobic MTA metabolism up through the point of volatile methanethiol release, consistent with a housekeeping function.

To assess methionine salvage and methanethiol formation from the MTA that is produced endogenously by normal cellular processes, cells were incubated with decreasing amounts of sulfate and the rate of methanethiol released by the cells was measured. Methanethiol was released by cells nearly 10-fold faster under sulfate-sufficient conditions (≥100 μM SO_4_^−2^) than under sulfate-limiting conditions (<100 μM SO_4_^−2^) (Table 2). This indicates that under conditions where sulfate is plentiful, a fraction of methanethiol produced from the metabolism of MTA was released into the environment. However, when the total amount of available sulfur became limiting, the vast majority of methanethiol produced by MTA metabolism was further metabolized, presumably by O-acetyl-l-homoserine sulfhydrylase, to generate l-methionine in order to maintain internal sulfur pools.

## DISCUSSION

Sulfur availability is imperative for cell viability, underscored by the fact that an estimated 1% to 2% of bacterial dry cell weight is composed of organic sulfur (32). To maintain proper intracellular sulfur pools in low-sulfur environments, many organisms employ salvage pathways for recycling sulfur-containing by-products arising from internal metabolic processes. In many organisms, the dead-end, toxic metabolite 5′-methyl-thioadenosine (MTA) is produced as a result of polyamine, homoserine lactone, ethylene, and betaine lipid biosynthesis. MTA may be subsequently metabolized (or salvaged) back to l-methionine by one of several pathways (Fig. 1). The recent discovery of several new bacterial methionine salvage pathways (MSPs) for the recycling of MTA in oxic and/or anoxic environments reveals that the populations are microbially diverse in salvaging mechanisms. For R. palustris, this organism not only possesses an oxygen-independent MTA-isoprenoid shunt (Fig. 6; blue) as evidenced by metabolite identification findings of this study (Fig. 3 and 5) but also utilizes a versatile pathway, linking MTA to 2-(methylthio)ethanol (Fig. 2B) as the key sulfur-containing intermediate in both an aerobic DHAP-methanethiol shunt and anaerobic DHAP-ethylene shunt MSP (Fig. 6; green). In the aerobic DHAP-methanethiol shunt, 2-(methylthio)ethanol is further metabolized to methanethiol, the direct precursor to methionine for sulfur salvage (Table 2) (Fig. 2A). In the anaerobic DHAP-ethylene shunt, 2-(methylthio)ethanol is further metabolized into ethylene and an unknown sulfur-containing intermediate that is presumably further recycled back to methionine (15). Thus, under aerobic conditions R. palustris is unique in using two distinct pathways, the MTA-isoprenoid shunt and the DHAP-methanethiol shunt, to metabolize MTA, each resulting in methanethiol production. In contrast, R. rubrum uses only the MTA-isoprenoid shunt under aerobic conditions. Under anaerobic conditions, R. palustris uses the DHAP-ethylene shunt MSP and likely the MTA-isoprenoid shunt as well (15, 19).

Utilization of a functional MTA-isoprenoid shunt by R. palustris signifies that this pathway is not solely specific to R. rubrum. At least 25 sequenced bacterial species were observed to possess homologues of the R. rubrum RLP and Cupin (>28% and >45% amino acid sequence identity, respectively), as well as putative MTA phosphorylase (MtnP) and 5-(methylthio)ribose-1-P isomerase (MtnA) genes, in a similar organization (14, 19). Of these, 13 are proteobacteria (e.g., R. rubrum, R. palustris sp., *Rhodomicrobium* sp., and *Nitrosococcus* sp.), while 9 are *Firmicutes* (Dialister sp. and Veillonella sp.) and 2 are *Deinococcus* (Meiothermus sp.).

The discovery here of an aerobic DHAP-methanethiol shunt MSP in which MtnP, MtnA, and 5-(methylthio)ribulose-1-P aldolase (Ald2) participate ascribes a general role for these three enzymes. Together, they catalyze the formation of 2-(methylthio)acetaldehyde from MTA as the key intermediate in at least two distinct methionine salvage pathways. Approximately 6.5% of all sequenced bacteria in the KEGG database possess homologues of these three enzymes, either with their genes all in the same operon, as in the case of R. rubrum, or with *mtnP* and *mtnA* together and *ald2* elsewhere, as in the case of R. palustris (15). With regard to habitat, these organisms reside mostly in terrestrial ecosystems; 43% of those species are soil sediment bacteria, as previously noted from a comprehensive genome analysis study analyzing these three key enzymes (15). Besides R. rubrum and R. palustris, it is unknown whether other organisms possessing MtnP, MtnA, and Ald2 also possess a functional aerobic DHAP-methanethiol shunt and/or anaerobic DHAP-ethylene shunt MSP. Therefore, identification of the methanethiol-catalyzing and ethylene-catalyzing enzymes will be of importance for elucidating the complete pathways.

### Role of R. palustris RuBisCOs and RuBisCO-like proteins in MTA metabolism.

Of all sequenced organisms, R. palustris is the only one reported to contain two different subclasses of RLPs as well as two bona fide RuBisCOs. Like its R. rubrum counterpart, the DeepYkr RLP (RLP1) of R. palustris was found to catalyze the requisite 5-(methylthio)ribulose-1-P 1,3-isomerization step of the MTA-isoprenoid shunt, indicating a potential common functionality for all RLP1-type enzymes, as this is currently the only known *in vivo* function for this subgroup of RLPs. Surprisingly, neither the RuBisCOs nor the Photo RLP (RLP2) appeared to participate by any means in MTA metabolism, either aerobically or anaerobically. Previous *in vitro* characterization of the R. palustris RLP2 indicated that it catalyzed a reaction in which only a small percentage of the substrate [5-(methylthio)ribulose-1-P] was converted to 1-(methylthio)xylulose-5-P after extended periods of time (22). It was concluded from this that R. palustris RLP2 might also participate in MTA metabolism in this organism. However, in this study, growth phenotypes (Table 1) and *in vivo* metabolite analysis of RLP2 deletion strains (Fig. 3), as well as *in vitro* kinetic rate determinations performed with recombinant enzymes, demonstrated that RLP2 plays a negligible role, if any, in the MTA-isoprenoid shunt and sulfur metabolism in general. Compared to the C. tepidum Photo RLP knockout strain (33), the R. palustris Photo RLP (RLP2) deletion strain does not present any of the observed pleiotropic effects found in C. tepidum, particularly with respect to phototrophic growth on various sulfur sources (25). This further highlights the mechanistic diversity that exists among the various subgroups of RLPs, indicative of their low amino acid sequence homology (25). Further experiments are required to elucidate the specific function of RLP2 in R. palustris.

### Role of multiple MTA metabolic pathways.

R. palustris is the first organism to have been observed to possess two aerobic MTA metabolic pathways. Interestingly, the MTA-isoprenoid shunt and DHAP-methanethiol shunt MSPs function simultaneously as evidenced by feeding experiments (Fig. 2B), and both result in the release of methanethiol as an intermediate (Fig. 2A and 3). Why R. palustris has two aerobic MSPs that converge to methanethiol and how the fate of 2-(methylthio)ethanol is different under aerobic conditions (DHAP-methanethiol shunt) from that under anaerobic conditions (DHAP-ethylene shunt) are unclear. That R. rubrum cannot metabolize 2-(methylthio)ethanol aerobically but can do so anaerobically (15) suggests that a different set of enzymes catalyzes the remaining unknown terminal steps of the DHAP-methanethiol and DHAP-ethylene shunt MSPs. Regardless, given that the MTA-isoprenoid shunt channels five carbon units of the MTA backbone into isoprenoid synthesis via DXP and that the DHAP-methanethiol and DHAP-ethylene shunt MSPs channel three carbon units of the MTA backbone into central carbon metabolism via DHAP, these pathways are poised to help R. palustris and similar organisms balance carbon pools in situations in which the MTA metabolism rate is elevated (e.g., polyamine synthesis, quorum sensing, betaine lipid metabolism, sulfate limitation [8, 10, 30]). Presumably, most of the DHAP generated during aerobic MTA metabolism enters into glycolysis for conversion into d-glyceraldehyde 3-phosphate, whereas the DHAP is likely used for ribulose-1,5-bisphosphate regeneration as part of the Calvin-Benson-Bassham cycle under anaerobic conditions.

### MTA-isoprenoid shunt and DHAP-methanethiol shunt as housekeeping pathways for metabolism of MTA to methanethiol.

Previous experiments performed with both R. palustris and R. rubrum indicated that ethylene production via the anaerobic DHAP-ethylene shunt was downregulated by the presence of excess sulfate (15). In contrast, neither the MTA-isoprenoid shunt nor the aerobic DHAP-methanethiol shunt appeared to be upregulated through the generation of methanethiol by the presence of saturating levels (≥100 μM) of sulfate (Table 2). This is consistent with earlier observations of R. rubrum (14) and observations here of R. palustris indicating that, regardless of the sulfur source used to grow cultures (MTA or sulfate), volatile methanethiol was liberated from the cells into the environment at the same rates upon feeding with MTA. In addition, the R. rubrum *mtnP*, *mtnA*, *rlp1*, *cupin*, and *ald2* genes all do not appear to be appreciably regulated by the presence of MTA under either aerobic or anaerobic conditions (19, 21). Altogether, these findings are consistent with a housekeeping role for these pathways with respect to the metabolism of MTA to methanethiol under aerobic conditions.

When the total available level of sulfur became limiting (<100 μM), the rate of methanethiol release by the cells decreased (Table 2). Under such conditions, sulfur is a valuable commodity and is presumably conserved to maintain cellular sulfur pools by metabolizing all available methanethiol into methionine via O-acetyl-l-homoserine sulfhydrylase. In R. rubrum, expression of the O-acetyl-l-homoserine sulfhydrylase genes (R. rubrum A0774 [Rru_A0774] and Rru_A0784) is upregulated over 10-fold in the presence of MTA (14, 19). R. palustris has four O-acetyl-l-homoserine sulfhydrylase homologues (RPA2350, RPA2364, RPA4251, and RPA4766), so this organism presumably may also use one or more of these enzymes to recycle methanethiol to methionine. In addition, under conditions where available sulfur is plentiful, excess methanethiol produced by MTA metabolism that is not required by the cell may be liberated into the environment as volatile methanethiol.

Methanethiol and dimethyl sulfide (DMS) are two of the most important volatile organic sulfur compounds in terms of the global sulfur cycle (34), and DMS is a contributing factor in numerous environmental functions, including global warming, acid precipitation, and cloud formation (35, 36). An average of 38 to 40 Tg of DMS is released into the atmosphere annually, making it the most abundant organic sulfur gas emitted globally (34). In marine surface waters, bacteria utilize dimethylsulfoniopropionate (DMSP), produced by unicellular algae as an osmolyte, resulting in the production of DMS and methanethiol by-products (37). DMSP demethylation pathways result in the production of methanethiol, which can be further utilized to form methionine or released into the environment. Cleavage of DMSP by DMSP lyase directly results in the formation of DMS. Some studies suggest that when cells have sufficient sulfur pools, DMSP degradation switches from demethylation to cleavage (38). While DMSP metabolism is the major marine source of methanethiol and DMS, it is increasingly evident that alternate routes may significantly contribute to their production by terrestrial ecosystems. Many bacteria, particularly those from soil ecosystems (up to 76%), possess a methanethiol-dependent DMS synthase (MddA), which transfers the methyl group from SAM to methanethiol to form DMS (39). Other thio-methyltransferase reactions in bacteria, catalyzing the methylation of hydrogen sulfide to methanethiol and methanethiol to DMS, have also been reported (34, 39). However, their distribution and role in environmental methanethiol and DMS production are not as well understood. Typically, it has been assumed or observed that methanethiol is produced either by a thiol-methyltransferase (34) or by the degradation of methionine via methionine gamma lyase (39, 40). The results of the current study reveal that the previously unidentified MTA-isoprenoid shunt and/or the MTA-DHAP shunt pathways may also contribute to the production of volatile methanethiol in the environment. It is likely that R. palustris and other organisms further utilize methanethiol produced by these pathways, either as a sulfur source or for the production of DMS. Indeed, R. palustris encodes a homologue of methanethiol *S-*methlytransferase (RPA2835), which catalyzes the conversion of methanethiol into dimethyl sulfide via transmethylation from SAM (39). It will be of interest to determine the contribution of these newly discovered methanethiol-generating pathways to the total levels of methanethiol and DMS in the environment as well as to denote the bacteria that employ these pathways.

## MATERIALS AND METHODS

### Bacterial strains and growth conditions.

R. palustris strains used in the current study are listed in Table 1 and were derived from wild-type strain CGA010. The R. rubrum wild-type strain (ATCC 11170), a RLP deletion strain (Δ*rlp*, strain “WR”), and a form II RuBisCO (*cbbM*) deletion strain with a *nifA* (M173V) mutation (Δ*cbbM* Δ*nifA*_M173V_; strain I19*) are described elsewhere (26, 41). E. coli strain SM10 (Biomedal Lifescience) was used for plasmid transfer via conjugative mating into R. palustris. E. coli strains BL21(DE3) (Millipore) with a pG-TF2 chaperone plasmid (TaKaRa) and Rosetta2(DE3) pLysS (Millipore) were used for recombinant protein synthesis.

All E. coli strains were grown aerobically in lysogeny broth (LB) media at 37°C with shaking at 280 rpm unless otherwise noted. All R. palustris strains were initially grown aerobically in PYE complex medium (0.3% peptone, 0.3% yeast extract, 10% Ormerod’s basal salts [42], 1 mg/liter thiamine, 1 mg/liter nicotinic acid, 15 μg/liter biotin) supplemented with 50 μg/liter streptomycin at 30°C with shaking at 280 rpm.

For growth studies, R. palustris cultures initially grown in PYE complex medium were washed aerobically three times in sulfur-free Ormerod’s malate minimal media (OMM) (26) containing 20 mM dl-malate, 1 mg/liter thiamine, 1 mg/liter nicotinic acid, 15 μg/liter biotin, and 15 μM 4-aminobenozic acid. Washed cells were used to inoculate 10 ml of OMM in either a capped 22-by-175-mm culture tube (aerobic growth studies) or a 20-by-145-mm sealed anaerobic culture tube (anaerobic growth studies) to an initial optical density at 660 nm (OD_660_) of ~0.03. Cultures were then supplemented with 500 μM ammonium sulfate or 500 μM MTA and incubated at 30°C either aerobically in the dark with shaking at 280 rpm or anaerobically in an incandescent lighted chamber (1,500 lx). R. rubrum growth studies were performed as previously reported (26).

### Generation of R. palustris deletion strains and complementation vectors.

In-frame nonpolar gene deletions were created by double homologous recombination using the pK18mobsacB suicide vector and sucrose selection, as previously reported (15, 43). Plasmids for deletion of R. palustris *rlp1*, *rlp2*, *ald2*, and *cupin* were as previously reported (15). For deletion of R. palustris RuBisCO form I (*cbbLS*) and form II (*cbbM*) genes, mutant strains were obtained as previously reported (44). Fragments were digested with appropriate restriction enzymes (New England Biolabs) and ligated into digested suicide vector pK18mobsacBst (15). The resulting plasmids were transformed into E. coli SM10 and transferred to R. palustris by conjugative mating. Transconjugants and double recombinants harboring the desired deletions were screened as previously described (15, 43). Antibiotics for transconjugant selection were kanamycin (50 μg/ml) and streptomycin (500 μg/ml).

Gene complementation of R. palustris deletion strains was performed via plasmid-based gene expression from the LacZ promoter using pBBR1-MCS5 (see Table S1 in the supplemental material). Plasmids pBBR1-RpMtnP (where "RpMtnP" represents "R. palustris MtnP"), pBBR1-RpMtnA, and pBBR1-RpAld2 were as previously reported (15) for gene expression of R. palustris *mtnP*, *mtnA*, and *ald*2, respectively. Conjugative transfer of plasmids via biparental mating between E. coli SM10 and R. palustris was performed as previously described (15). Transconjugants were selected using streptomycin (500 μg ml^−1^).

10.1128/mBio.00407-18.9 TABLE S1  Plasmids used in this study. Download TABLE S1, DOCX file, 0.1 MB. Copyright © 2018 Miller et al. 2018 Miller et al. This content is distributed under the terms of the Creative Commons Attribution 4.0 International license.

### Identification of [methyl-^14^C]MTA-derived metabolites.

All [methyl-^14^C]-labeled standards were synthesized from [methyl-^14^C]*S-*adenosyl-l-methionine (PerkinElmer) as previously reported (19). R. palustris strains were grown aerobically in 50 ml of sulfur-free OMM supplemented with 200 μM MTA to an OD_660_ of ~0.4. Cells were washed twice with sulfur-free OMM and resuspended to a final OD_660_ of ~8.0 to 10.0 in 2.5 ml of sulfur-free OMM as previously reported (14). Cells were fed with 20 μM MTA and 30 μM [methyl-^14^C]MTA. Cell suspensions were placed in 5-ml conical glass vials and bubbled with compressed filtered air while being shaken at 60 rpm in a 30°C water bath. For detection of methanethiol, 500 μM DTNB was also added to the media. Time point data were acquired by pelleting a 300-μl cell suspension, separating cells from the media by centrifugation, and storing at −80°C.

For detection of [methyl-^14^C]-labeled 2-(methylthio)ethanol and methanethiol-DTNB adduct, metabolites present in the media were separated by Zorbax C_18_ reverse-phase chromatography (Agilent) at 30°C with a flow rate of 0.8 ml/min on a Shimadzu Prominence HPLC system with an inline UV light-visible light (UV-Vis) detector (215 nm, 260 nm, and 320 nm) and a β-RAM radiometric detector (IN/US Systems). Metabolites were eluted on a gradient composed of buffer A with 0.2% buffer B for 5 min, 0.2% to 50% buffer B for 23 min, 50% to 100% buffer B for 14 min, and 100% buffer B for 3 min, followed by reequilibration with 0.2% buffer B for 12 min (buffer A, 20mM ammonium acetate; buffer B, 20 mM ammonium acetate and 50% acetonitrile; pH to 6.8 using acetic acid).

For detection of [methyl-^14^C]-labeled (methylthio)pentose-phosphate sugars, cell supernatants were concentrated by vacuum centrifugation to 100 μl and 400 µl of acetonitrile was added. Cell pellets were subsequently extracted twice with 250 μl of this mixture by subjecting cells to vortex mixing for 5 min at room temperature (RT) followed by incubation at −20°C for 15 min and centrifugation to remove cell debris. Initially, extracted metabolites were separated as previously described (14) by HILIC-HPLC on a SeQuant ZIC-pHILIC column (Millipore) using UV-Vis detection (215 nm and 260 nm) and an inline radiometric detector (Fig. 5) as described above. Separation of metabolites was optimized using a modified gradient as follows: 100% buffer B for 5 min, 100% to 40% buffer B for 40 min, and 40% to 100% buffer B for 10 min, followed by reequilibration with 100% buffer B for 15 min. Coordinately, the flow rates were 0.35 ml/min for 15 min, 0.35 to 0.25 ml/min for 2 min, 0.25 ml/min for 28 min, and 0.25 to 0.35 ml/min for 2 min, followed by 0.35 ml/min for 23 min (buffer A, 20 mM ammonium bicarbonate; buffer B, 20 mM ammonium bicarbonate and 80% acetonitrile; pH 9.2 using ammonium hydroxide).

### Methanethiol release measurements.

R. palustris strains were grown aerobically in 50 ml of sulfur-free OMM supplemented with 500 μM MTA or 500 μM sulfate to an OD_660_ of 0.3 to 0.5. Cells were washed twice with sulfur-free OMM and resuspended to a final OD_660_ of 3.0 to 5.0 in 5 ml sulfur-free OMM as previously reported (14). Cells were fed with 1 mM MTA, and Ellman’s reagent [5,5′-dithiobis-(2-nitrobenzoic acid) (DTNB)] was added to reach 500 μM. Cells were incubated at 30°C with shaking at 280 rpm for 15 h, after which 250 μl of cell suspension was centrifuged to collect the media. Released methanethiol, captured as a DTNB adduct (MT-DTNB), was separated by reverse-phase HPLC as described above. MT-DTNB was detected at 360-nm-wavelength absorbance and quantified based on an MT-DTNB standard calibration curve.

### Gene expression and purification of recombinant proteins.

Plasmids for expression of the Bacillus subtilis *mtnK* and *mtnA* genes and of the R. rubrum *rlp1* and *ald2* genes were as previously reported (14). Expression plasmids for R. palustris *rlp1* and *rlp2* genes and E. coli DXP reductoisomerase (*DRI*) genes (Table S1) were created by amplifying each gene via PCR using primers with restriction enzyme sites listed in Table S2 and cloning into pET28 (Millipore). B. subtilis *mtnK* and *mtnA*, R. rubrum *rlp*, and E. coli *dri* were expressed for 3 h in strain Rosetta2(DE3) pLysS grown in LB media at 37°C with shaking at 280 rpm supplemented with kanamycin (50 μg/ml), chloramphenicol (34 μg/ml), and 1 mM IPTG (isopropyl-β-d-thiogalactopyranoside). Proteins were purified as previously described (44). To increase protein solubility, E. coli BL21(DE3) pG-TF2 was used for expression of *rlp1* and *rlp2*. Cells were initially grown at 37°C with shaking at 280 rpm to an OD_660_ of ~0.5 in LB media supplemented with kanamycin, chloramphenicol, and tetracycline (2 μg/ml). Cells were cold shocked on ice for 30 min and supplemented with 125 μM IPTG, and expression proceeded for 12 h at 15°C with shaking at 180 rpm. Due to high coexpression of chaperone proteins, RLP1 and RLP2 were purified by a linear gradient (10 column volumes) of 20 to 250 mM imidazole in 300 mM NaCl–50 mM Tris-Cl (pH 7.5) on a 10-ml column of nickel-nitrilotriacetic acid (Ni-NTA) agarose (Qiagen). Purified proteins were buffer exchanged into a mixture of 20 mM Tris-Cl (pH 7.5), 1 mM EDTA (pH 8.0), 1 mM dithiothreitol (DTT), and 300 mM NaCl by the use of a centrifugal concentrator (Amicon, Millipore). Size exclusion chromatography was performed at 4°C using a Superose 12 column (GE Life Science) equilibrated with the buffer described above at 0.5 ml/min. Glycerol was added to reach a level of 10%, and proteins were stored at −80°C. Protein concentrations were determined by UV absorbance, and purity was assessed by SDS-PAGE (Fig. S7).

10.1128/mBio.00407-18.10 TABLE S2  Primers used in this study. Download TABLE S2, DOCX file, 0.1 MB. Copyright © 2018 Miller et al. 2018 Miller et al. This content is distributed under the terms of the Creative Commons Attribution 4.0 International license.

### Enzyme assays.

The activity and kinetics of the RLP enzymes were determined by a coupled spectrophotometric assay following the oxidation of NAPDH at a 340-nm wavelength using a spectrophotometer (Carey, Varian). 5-(Methylthio)ribulose-1-P was produced *in situ* using 0.5 mM 5-(methylthio)ribose, 1 mM ATP, 2.5 mM MgCl_2_, 10 μM B. subtilis MtnK, and 10 μM MtnA. R. rubrum or R. palustris RLP was added to 0 to 300 nM, and production of 1-(methylthio)xylulose-5-P was monitored by adding 10 μM R. rubrum Cupin, 10 μM E. coli DRI, 1 mM DTT, 1 mM MnCl_2_, and 0.4 mM NADPH.

Identification of specific products of the RLP enzymes with 5-(methylthio)ribulose-1-P as the substrate was accomplished by HILIC. 5-(Methylthio) ribose (1 μM) and [methyl-^14^C]5-(methylthio)ribose (50 μM) were added to a reaction mixture containing 10 mM MgCl_2_, 50 mM HEPES (pH 7.5), 5 mM ATP, 2 mM DTT, 20 μM B. subtilis MtnK, 10 μM B. subtilis MtnA, and 10 μM RLP in a 50-μl total volume. Reaction mixtures were incubated at 30°C for 2 h, acetonitrile was added to reach a 300-μl total volume, and then the reaction mixture was separated by HILIC-HPLC with UV detection at a 215-nm wavelength and in-line radiometric detection as described above. Specific production of 1-(methylthio)xylulose-5-P was further confirmed by adding to the reaction mixtures described above 2 mM DTT, 1 mM MnCl_2_, and 10 μM R. rubrum cupin. Reactions were stopped by addition of 10 mM DTNB, and [methyl-^14^C]methanethiol as a DTNB adduct was resolved by reverse-phase HPLC with UV detection at a 320-nm wavelength and in-line radiometric detection as described above.
