# Supplementary figures and images for: Two Distinct Aerobic Methionine Salvage Pathways Generate Volatile Methanethiol in Rhodopseudomonas palustris
Source: mBio. 2018 Apr 10;9(2):e00407-18. doi: 10.1128/mBio.00407-18 (PMC5893883; doi:10.1128/mBio.00407-18)

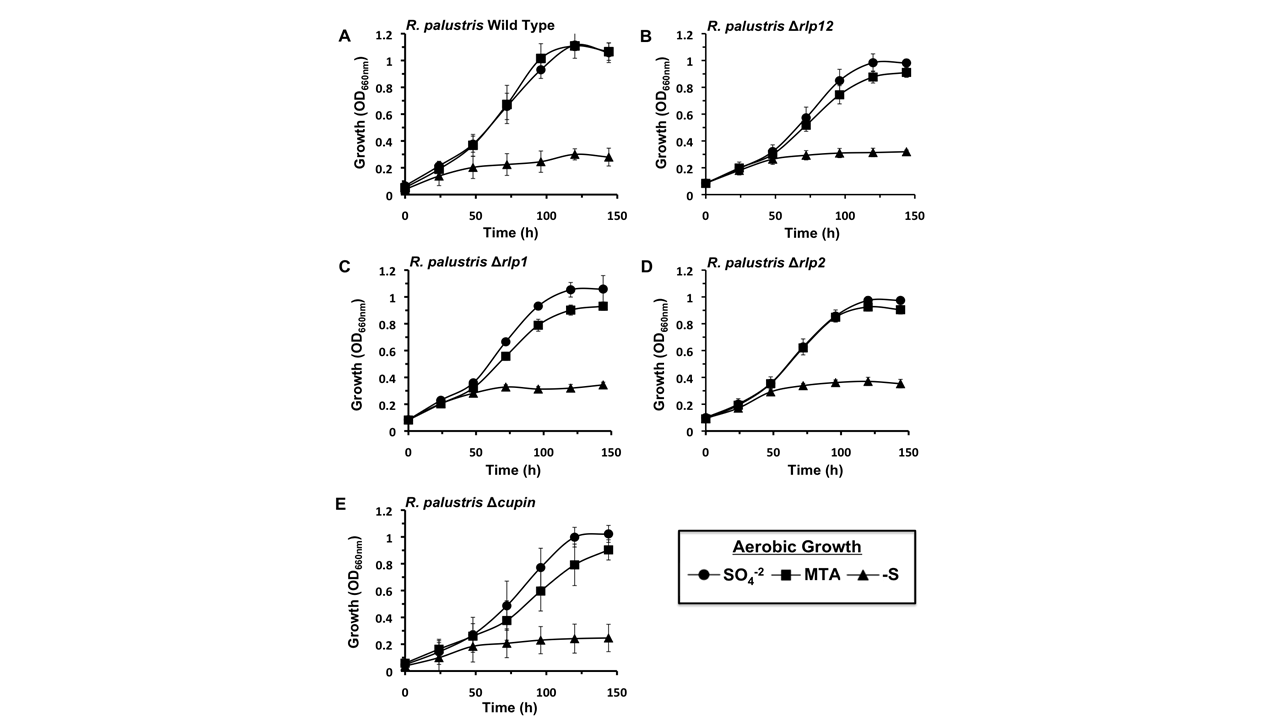

Supplement: FIG S1 [file mbo001183820sf1.tif]

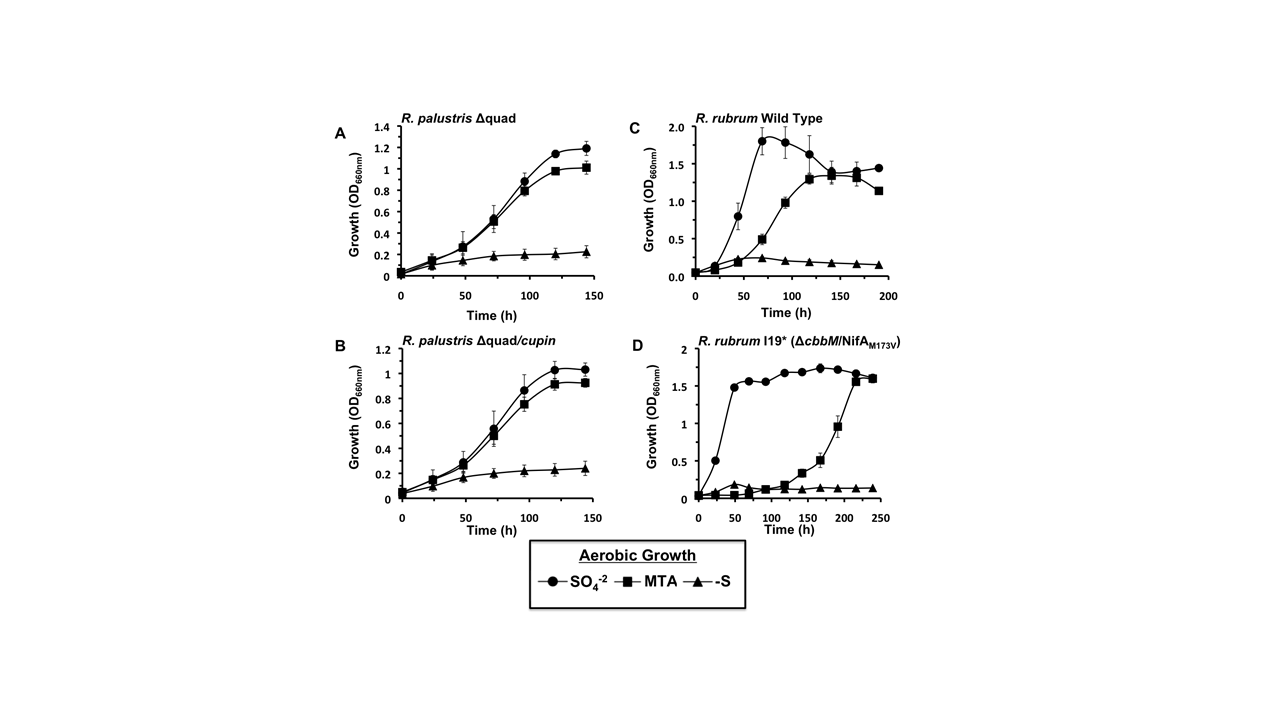

Supplement: FIG S2 [file mbo001183820sf2.tif]

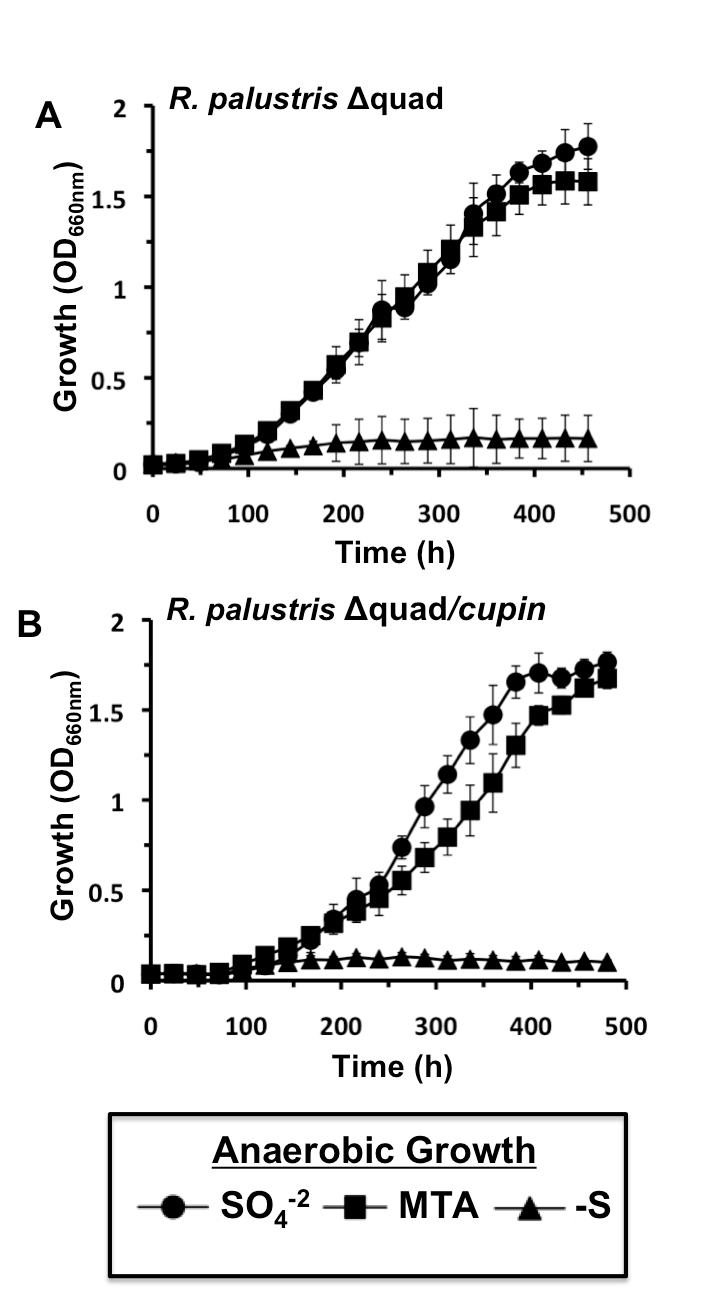

Supplement: FIG S3 [file mbo001183820sf3.tif]

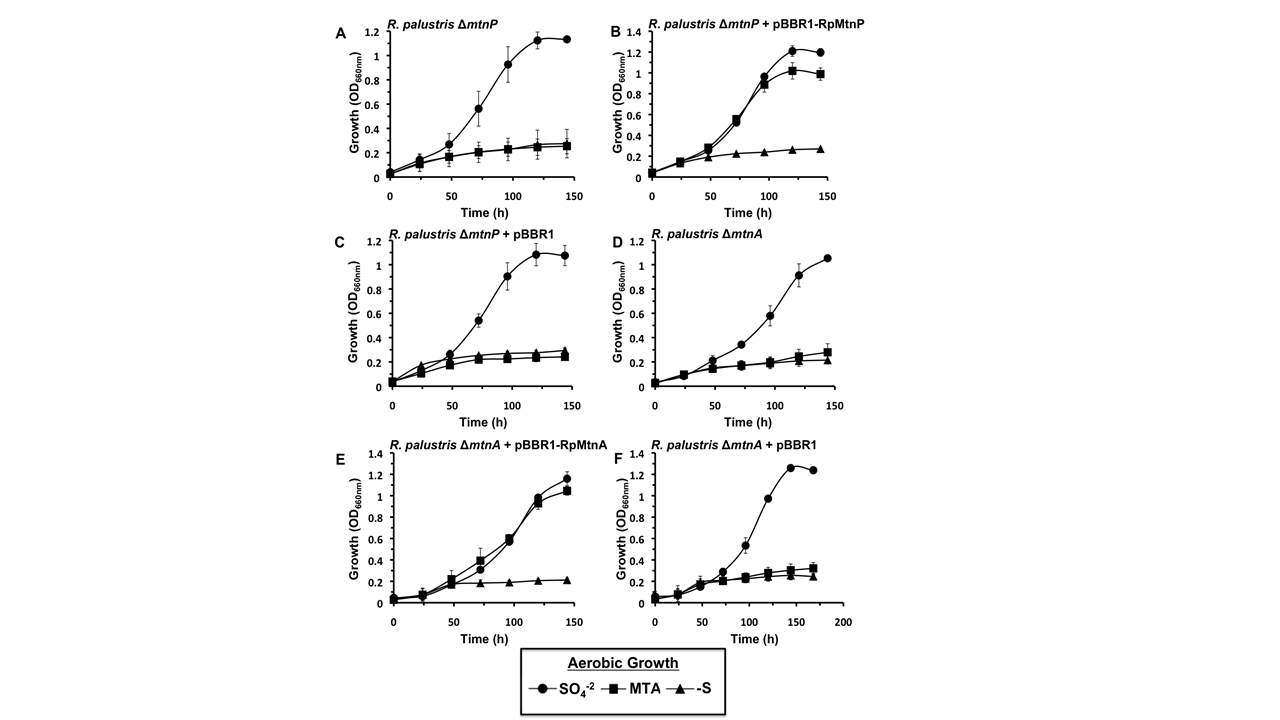

Supplement: FIG S4 [file mbo001183820sf4.tif]

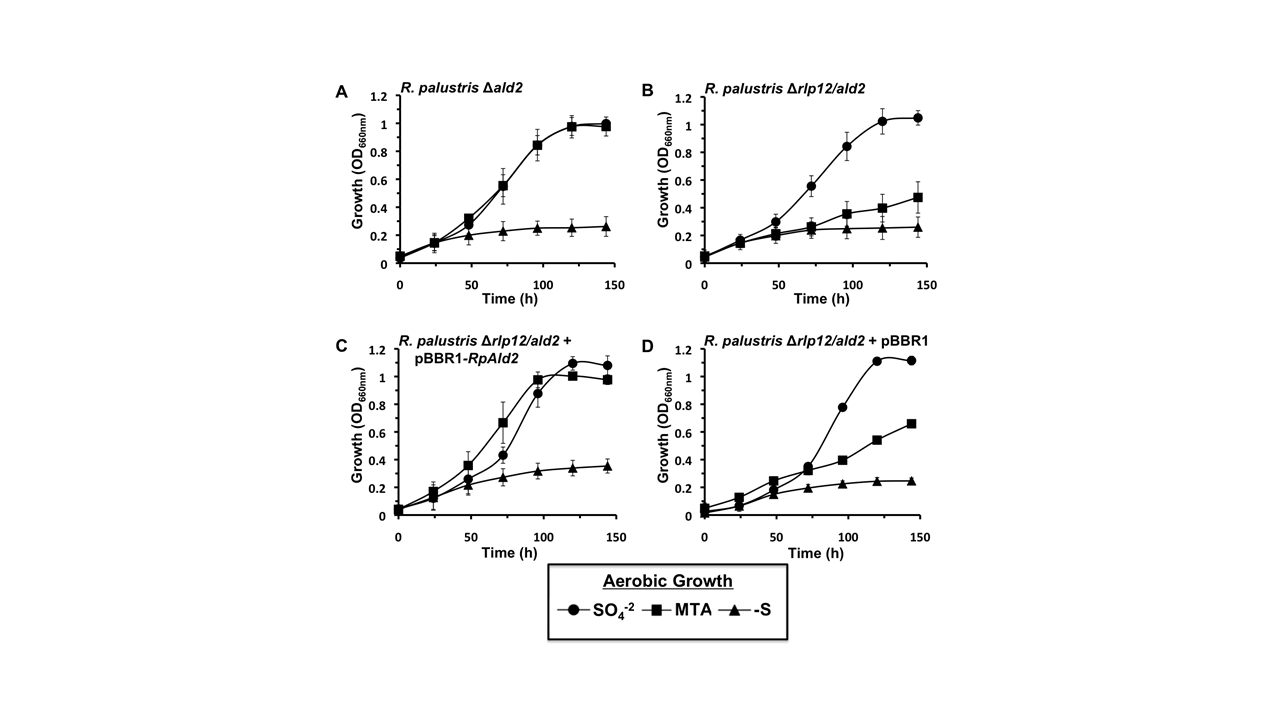

Supplement: FIG S5 [file mbo001183820sf5.tif]

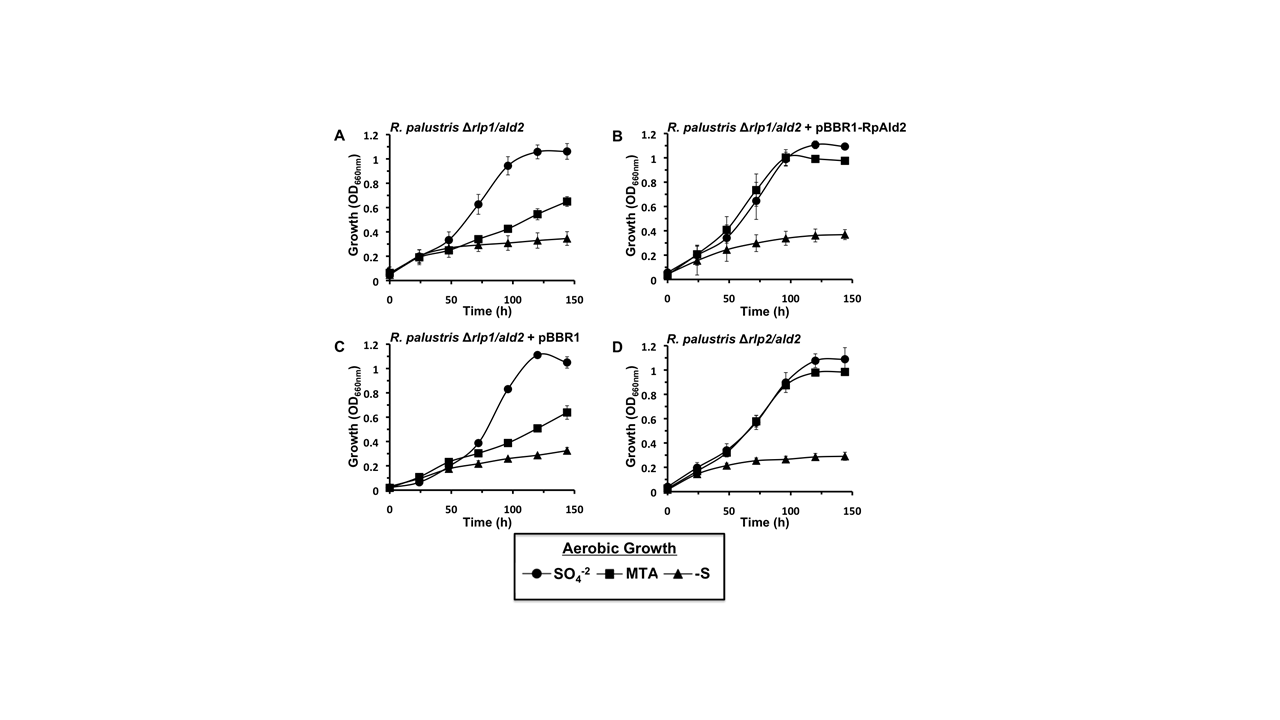

Supplement: FIG S6 [file mbo001183820sf6.tif]

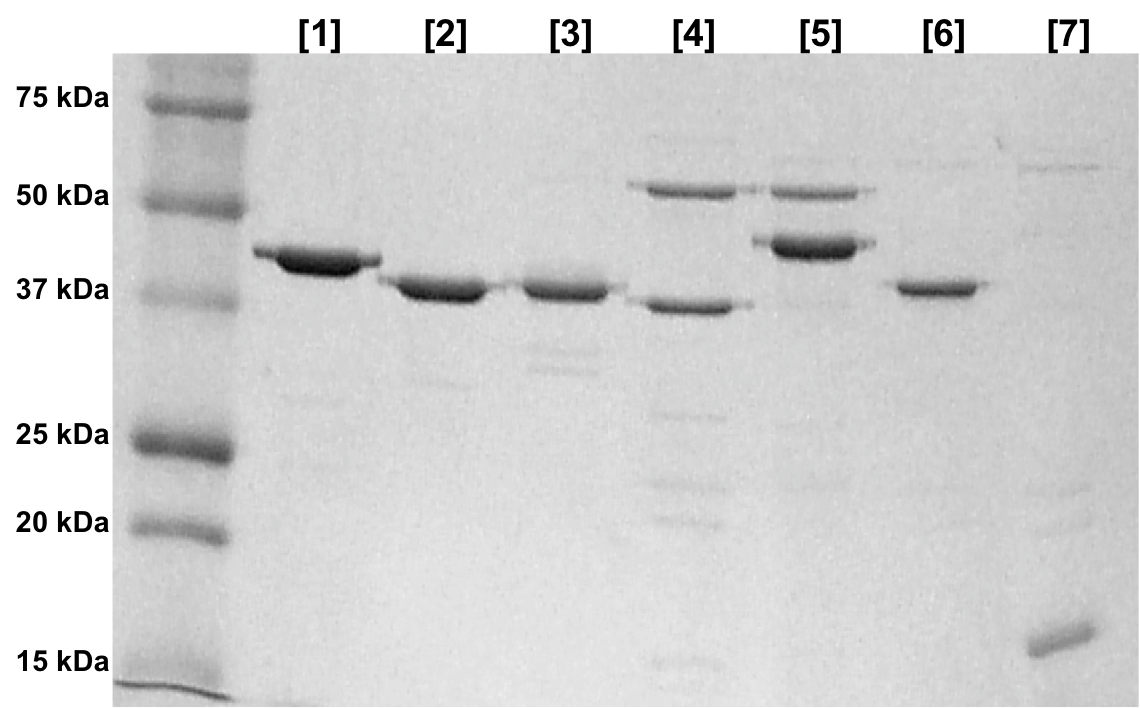

Supplement: FIG S7 [file mbo001183820sf7.tif]

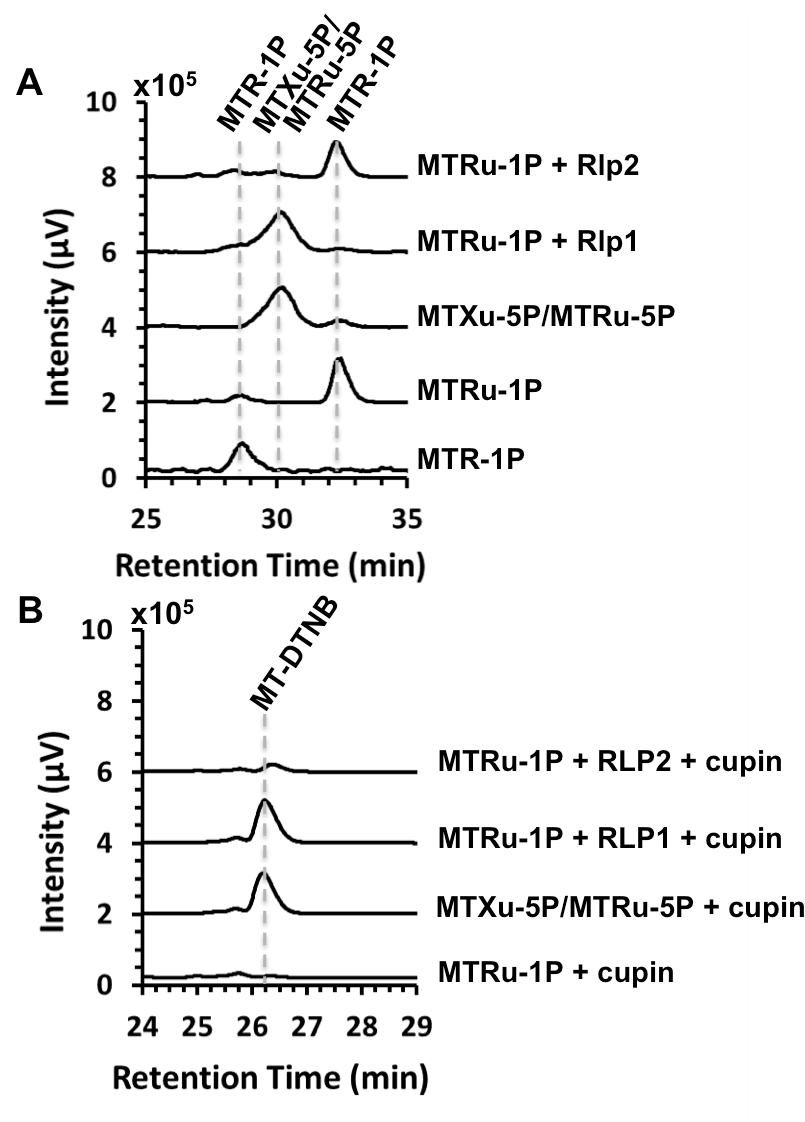

Supplement: FIG S8 [file mbo001183820sf8.tif]

**
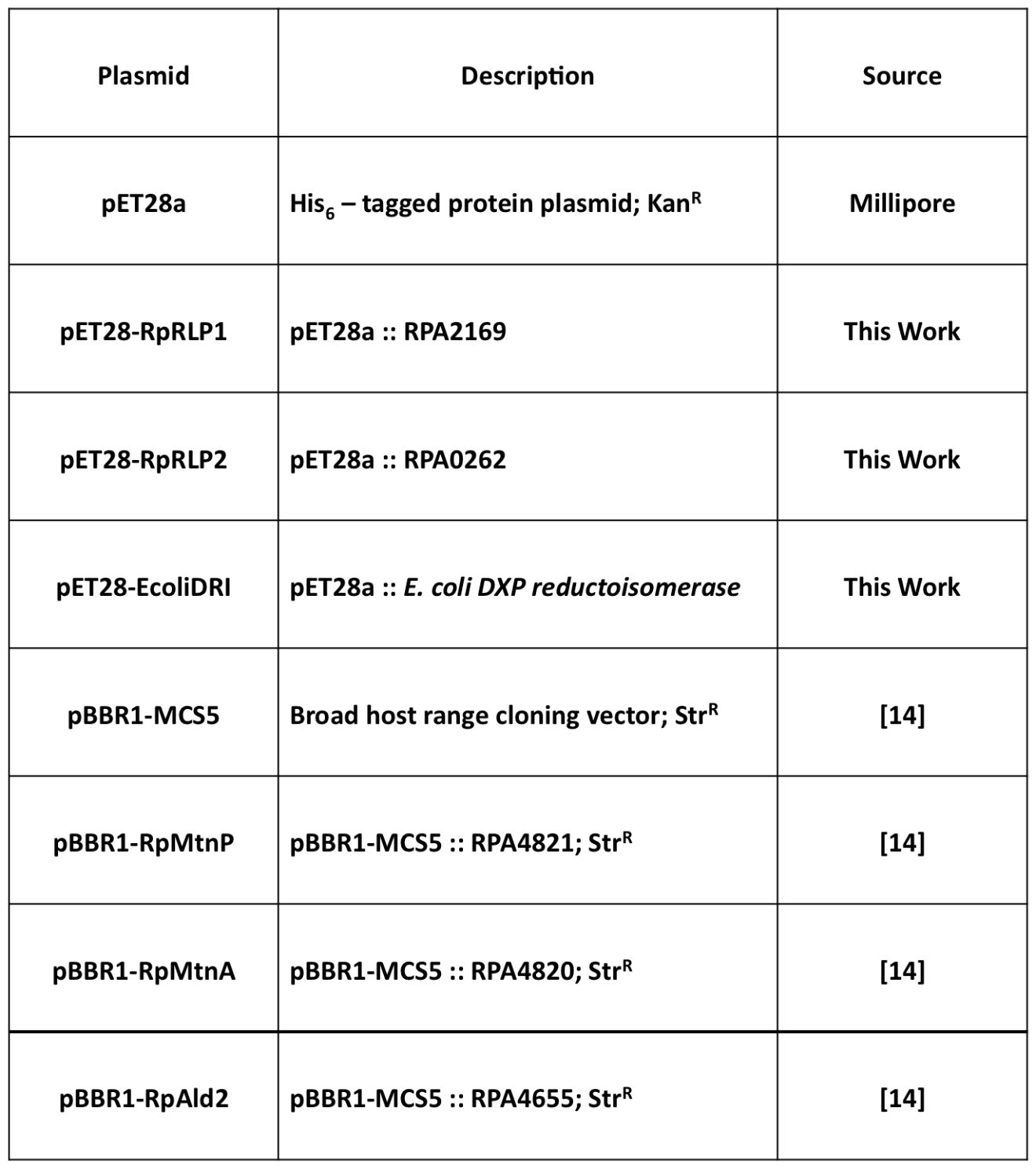
**

Supplement: TABLE S1 [file mbo001183820st1.docx]
