# Supplementary material for: Two Distinct Aerobic Methionine Salvage Pathways Generate Volatile Methanethiol in Rhodopseudomonas palustris
Source: mBio. 2018 Apr 10;9(2):e00407-18. doi: 10.1128/mBio.00407-18 (PMC5893883; doi:10.1128/mBio.00407-18)
Supplement: TABLE S2 [file mbo001183820st2.docx]

**Supp. Table 2.** Primers used in this study


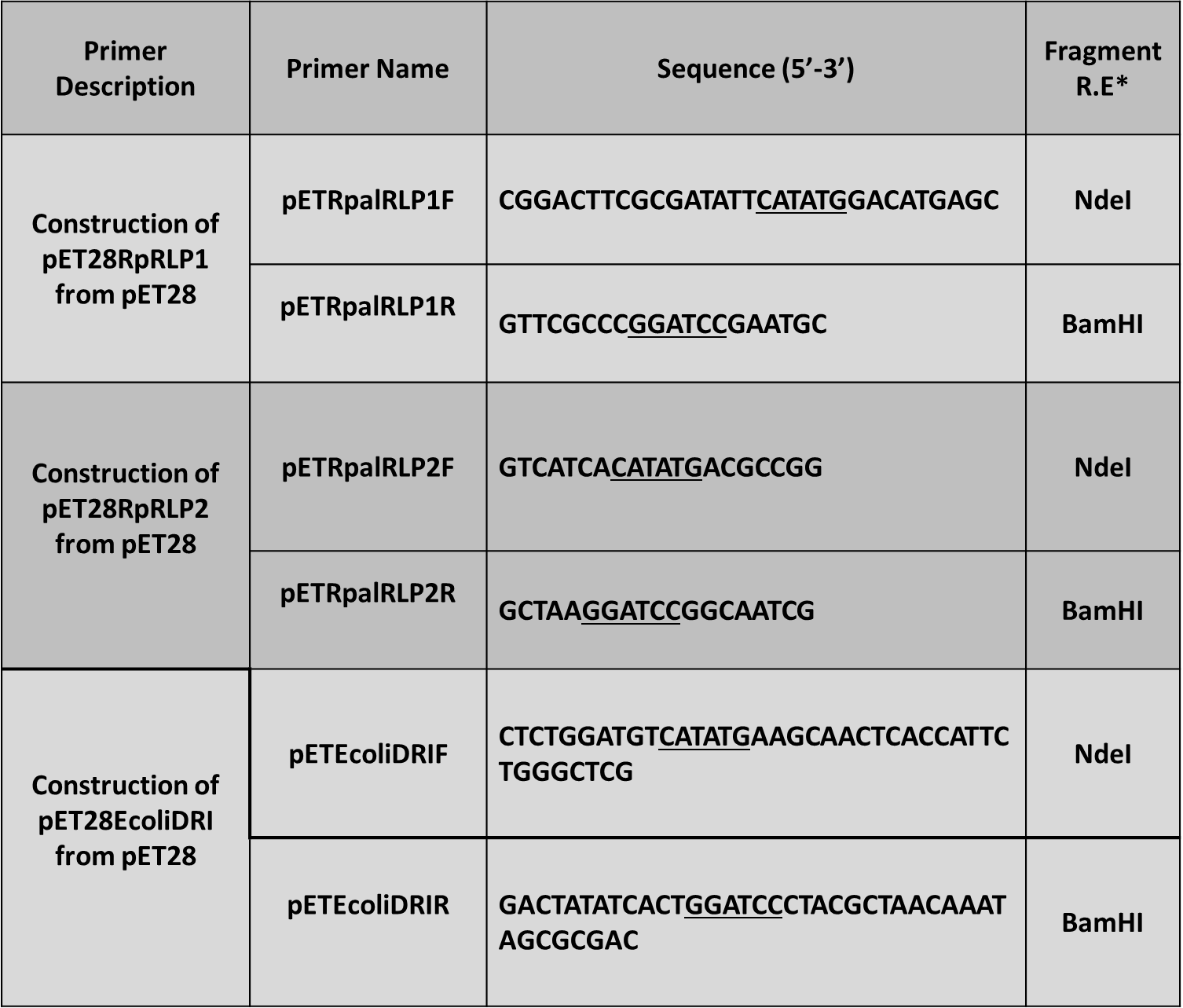


* Restriction enzyme used to digest PCR fragment amplified using indicated primer
